# Public perceptions during the first wave of the COVID-19 pandemic in Canada: a demographic analysis of self-reported beliefs, behaviors, and information acquisition

**DOI:** 10.1186/s12889-022-13058-3

**Published:** 2022-04-09

**Authors:** Jeanna Parsons Leigh, Rebecca Brundin-Mather, Andrea Soo, Emily FitzGerald, Sara Mizen, Alexandra Dodds, Sofia Ahmed, Karen E. A. Burns, Kara M. Plotnikoff, Bram Rochwerg, Jeffrey J. Perry, Jamie L. Benham, Kimia Honarmand, Jia Hu, Raynell Lang, Henry T. Stelfox, Kirsten Fiest

**Affiliations:** 1 School of Health Administration, Faculty of Health, Dalhousie University, Halifax, Nova Scotia Canada; 2 Department of Critical Care Medicine, Cumming School of Medicine, University of Calgary, Calgary, Alberta Canada; 3 Department of Medicine and Libin Cardiovascular Institute, University of Calgary, Calgary, Alberta Canada; 4 Interdepartmental Division of Critical Care Medicine, University of Toronto, Toronto, Ontario Canada; 5 Unity Health Toronto – St. Michael’s Hospital, Toronto, Ontario Canada; 6 Li Ka Shing Knowledge Institute, St. Michael’s Hospital, Toronto, Ontario Canada; 7 Department of Health Research Methods, Evidence & Impact, McMaster University, Hamilton, Ontario Canada; 8 Department of Medicine, Division of Critical Care, McMaster University, Hamilton, Ontario Canada; 9 Department of Emergency Medicine, University of Ottawa, Ottawa, Ontario Canada; 10 The Ottawa Hospital Research Institute, Ottawa, Ontario Canada; 11 Department of Community Health Sciences, Cumming School of Medicine, University of Calgary, Calgary, Alberta Canada; 12 Department of Medicine, Cumming School of Medicine, University of Calgary, Calgary, Alberta Canada; 13 Division of Critical Care, Department of Medicine, Western University, London, Ontario Canada; 14 O’Brien Institute for Public Health, Cumming School of Medicine, University of Calgary, Calgary, Alberta Canada

**Keywords:** COVID-19, Coronavirus, Perception, Public health, Surveys and questionnaires, Pandemics, Information dissemination

## Abstract

**Introduction:**

We explored associations between sociodemographic factors and public beliefs, behaviors, and information acquisition related to the coronavirus disease 2019 (COVID-19) to identify how the experiences of subpopulations in Canada may vary.

**Methods:**

We administered a national online survey through Ipsos Incorporated to adults residing in Canada. Sampling was stratified by population age, sex, and regional distributions. We used descriptive statistics to summarize responses and test for differences based on gender, age, educational attainment, and household income using chi-squared tests, followed by weighted logistic regression.

**Results:**

We collected 1996 eligible questionnaires between April 26th and May 1st, 2020. Respondents mean age was 50 years, 51% were women, 56% had a post-secondary degree, and 72% had a household income <$100,000. Our analysis found differences within the four demographic groups, with age effects most acutely evidenced. Respondents 65 years and older were more likely to perceive the pandemic as very serious, less likely to report declines in overall health, and more likely to intend to get vaccinated, compared to 18–29 year olds. Women overall were more likely to report negative outcomes than men, including stress due to the pandemic, and worsening social, mental/emotional, and spiritual health. Respondents 45 and older were more likely to seek and trust information from traditional Canadian news sources, while 18-29 year olds were more likely to seek and trust information on social media; overall, women and respondents with a post-secondary degree were more likely to access and trust online information from public health sites.

**Conclusion:**

This study found important demographic differences in how adults living in Canada perceived the COVID-19 pandemic, the impacts on their health, and their preferences for information acquisition. Our results highlight the need to consider demographic characteristics in tailoring the format and information medium to improve large scale acceptance and uptake of mitigation and containment measures.

**Supplementary Information:**

The online version contains supplementary material available at 10.1186/s12889-022-13058-3.

## Background

The emergence of the novel severe acute respiratory syndrome coronavirus 2 (SARS-CoV-2) has led to a proliferation of rapidly evolving information about the coronavirus disease 2019 (COVID-19) and strategies to mitigate virus spread [1]. As a result, the public has been inundated with frequently updated information from varied sources [2, 3], and regularly tasked with deciphering fact from fiction [4]. Variable compliance with non-pharmaceutical interventions (NPIs) (eg, physical distancing, masking, business and public institution restrictions) due to information overload [1], consumption of misinformation [5], lack of awareness of recommendations [6, 7], and pandemic fatigue [8] all risk the effectiveness of NPIs [9]. It is critical that the public has timely and reliable access to accurate and consistent information about the pandemic in order to adapt their behaviours in ways that promote public health and safety [5, 10].

Canada is the second largest land area in the world, bordering north of the United States of America, with a population of 38 million, less than 1 % of the global population. The latest national census (2016) reported a median total income of $35,200CAD for a single-person household and $88,249CAD for a multi-person household before taxes [11]. While Canada’s progressive tax system contributes to improved income distribution, the gender pay gap is still evident as women on average earn 76.8 cents for every dollar earned by men [12].

Canada’s first case of COVID-19 was reported on January 27, 2020, and the first case of community transmission was reported 5 weeks later on March 5, 2020. Provinces and territories began instituting NPIs in varying degrees with rising COVID-19 case numbers. At the peak of the first wave of infections in Canada (about April 22^,^ 2020) there were 43,600 confirmed cases, with a 7-day rolling average daily infection rate of about 1900 and death rate of about 160 [13]. Canada experienced two subsequent waves of infection [14], and a fourth began in September 2021. At the time of publication submission (December 15, 2021) there were 1,858,000 COVID-19 cases (4858 cases per 100,000 people) and 30,000 deaths (78 per 100,000) in Canada [13].

Between April 26th and May 1st, 2020, at the peak of the first wave of COVID-19 in Canada, we conducted a national survey of self-reported perceptions of adults living in Canada, including their beliefs (e.g., concerns), behaviours (e.g., engagement in NPIs), and information acquisition (e.g., COVID-19 information navigation) [15]. Aggregate data from our survey highlighted the perceived negative impact of COVID-19 on personal health and healthcare safety and access. The data also showed that adults largely obtained and trusted information about COVID-19 from domestic news sources, however, adults from the region with the highest burden of COVID-19 cases (Québec) reported the least trust in the government and domestic news sources, and reported that they were significantly less intent on being vaccinated [15]. Our findings supported the importance of tailoring public health messaging to jurisdictions.

There is growing evidence documenting disparities in COVID-19 risk factors and disease outcomes based on race and ethnicity [16–20], sex [20–22], age [20, 23], housing [17, 18], and income [17, 24]. Data collected during previous infectious disease outbreaks have also reported sociodemographic differences amongst the public in knowledge [25] and behaviour [26–29]. Similarly, research early in the COVID-19 pandemic demonstrated that women and those of older age were more likely to report adhering to the Center for Disease Control and Prevention (CDC) guidelines [30]. Age and gender-based differences in accuracy of knowledge about COVID-19 were also reported, with older adults and women knowing more about COVID-19 and NPIs than younger adults and men [31]. However, there is limited data on socio-demographic variables that may be associated with differences in how the public is accessing, evaluating, and by extension, using information to make everyday life decisions in the context of a global pandemic [32]. To bridge this gap in the literature, we conducted a sub-study using our national survey data to explore associations between sociodemographic factors and public beliefs and information acquisition related to COVID-19 in Canada. We aimed to determine important variations in perceptions and preferences within broad population groups that will support the development of high-quality, tailored public health communications in Canada.

## Methods

We developed a cross-sectional, online, anonymous survey and contracted Ipsos Incorporated (https://www.ipsos.com/en-ca), an international research and polling firm, to administer the survey across Canada. All methods were performed in accordance with the guidelines and regulations of the Research Ethics Boards of Dalhousie University (#2020-5121) and the University of Calgary (#20-0538) who granted ethical approval for this study. Informed consent was sought from the participants completing the questionnaire. Prior to entering the survey, respondents reviewed an informed consent page; consent was implied by completing the survey.

### Questionnaire design

Using standard survey methodologies [33], we iteratively designed and pre-tested a questionnaire that ultimately consisted of 46 questions capturing three overarching domains of inquiry: beliefs (e.g., concerns about contracting the virus, changes in personal health, intent to vaccinate), information acquisition (e.g., knowledge of virus transmission, information sources accessed and trusted, misinformation identification), and prevention behaviors (e.g., self-isolation, physical distancing). Questions contained variable response options including 5-point and 7-point Likert scales, single-response multiple choice, and multiple-response checkboxes. We categorized the calculated difference in respondents’ retrospective ratings on a 5-point scale of five domains of overall health (mental/emotional, physical, social, economic, spiritual) at the start of 2020 to ratings of their current health as ‘worse’, ‘same’, or ‘better’. We included 21 questions to identify respondent demographics (e.g., sex assigned at birth, self-identified gender, age, ethnicity, marital status, education, income, children) and other personal characteristics (e.g., employment, political affiliation, religion, residence, health status). The final questionnaire was formatted in English and French. See Additional file 1 for a description of the survey development and an English survey copy.

### Questionnaire administration

We distributed the survey through Ipsos’ proprietary iSay panel of approximately 250,000 Canadians recruited through random-digit dialing telephone, email, and social networking websites. Panelists were eligible to participate if they were adults (≥18 years), lived in Canada, were able to read English or French, and were able to provide informed consent. No additional inclusion or exclusion criteria were applied. Sampling quotas were established and respondents screened based on age (18-34, 35-55, > 55), sex assigned at birth (female/male), and provincially defined regions (British Columbia, Alberta, Saskatchewan/Manitoba, Ontario, Québec, and Atlantic provinces) to ensure a representative sample of the Canadian population according to 2016 census data [11]. Once sampling quotas were met, data collection ceased. Respondents received Ipsos reward points after completing the survey; points are accumulated and redeemed for gift cards and merchandise.

### Sample size calculations

We derived a minimum sample size estimate of 385 using a standard survey sample size calculation (assuming an observed proportion of respondents selecting a specific response option of 50%) that incorporated population size (~ 36.3 million in Canada) [11], a 95% confidence level and a margin of error of 5%. We elected to collect 2000 survey responses to allow for subgroup analyses and calculated the associated margin of error to be +/− 2.2% at a 95% confidence level.

### Data analysis

We used descriptive statistics (frequencies (percent) or means (standard deviation)) to summarize respondent characteristics. Responses were weighted by age and sex census data [11]. We reported Likert scales as frequencies and percentages for each point on the scale and tested for significant associations within four primary demographic groups using Chi-squared tests of independence. The demographic groups included: (1) self-identified gender (woman; man); (2) age (18-29 years; 30-44 years; 45-64 years; 65 years and older); (3) education (high school diploma or less (high school); trade or vocational certification or some post-secondary college or university (trade/some post); post-secondary college or university degree (degree)); and (4) annual household income (<$50,000 (<$50 K); $50,000-$99,999 ($50 K- < $100 K); $100,000 or more ($100 K+)). Respondents who could not be classified into these categories were not included in the analyses. Regional differences in survey responses are reported elsewhere [15]. This study focused specifically on questions related to self-report health and information access and trust. We did not make adjustments for multiple comparisons as our objective was to explore for possible associations rather than confirm a priori hypotheses [34]. We conducted post-hoc comparisons using weighted univariate logistic regression to quantify sub-group differences within each demographic group based on Chi square test *p*-values less than 0.05. The logistic regression results are reported on p-values less than 0.001. The Odds Ratios (OR) reference groups were *man* (gender), *18-29 years* (age), *high school* (education), and *< $50 K* (household income). We conducted quantitative data analyses using R, version 3.5.1 [35]. The R package “survey” [36] was used to obtain weighted descriptive statistics, version 3.36. Statistical significance was set at α = 0.05.

## Results

We collected 1996 eligible questionnaires between April 26 to May 1, 2020. Survey respondents were on average 50 years old (Table 1) distributed across age categories reflective of the latest population census [11]. Just over half of respondents (*n* = 1080; 54.3%) were women, 56% (*n* = 1104) had completed a college or university degree, and close to three-quarters (*n* = 1171; 67.3%) reported a household income under $100,000 CAD (Table 1). A full summary of respondent characteristics is in STable 1, Additional file 2 . Pairwise comparisons of our primary demographic variables are in SFigure 1, Additional file 2.

**Table 1:** Participant demographics

Participant Characteristics	Number (%)^**a**^
**Gender** (*n* = 1979)	
Woman/girl	1080 (54.6)
Man/boy	899 (45.4)
**Age (in years)** (*n* = 1996)	
Mean (SD)	50 (34-66)
18-29	303 (15.2)
30-44	505 (25.3)
45-64	637 (31.9)
65+	551 (27.6)
**Highest Education** (*n* = 1975)	
High school equivalent, or less	396 (20.1)
Trade or technical college; some college/university	475 (24.1)
College/University/Postgraduate degree	1104 (55.9)
**Total Household Income** (*n* = 1741)	
0$ - $49,999	600 (34.5)
$50,000 - $99,999	658 (37.8)
$100,000 or more	513 (29.5)

^a^Frequencies and percent are noted unless otherwise indicated. Prefer not to answer response options are excluded from data analyses and individual *n* reported

### Health and prevention

**Fig. 1:**
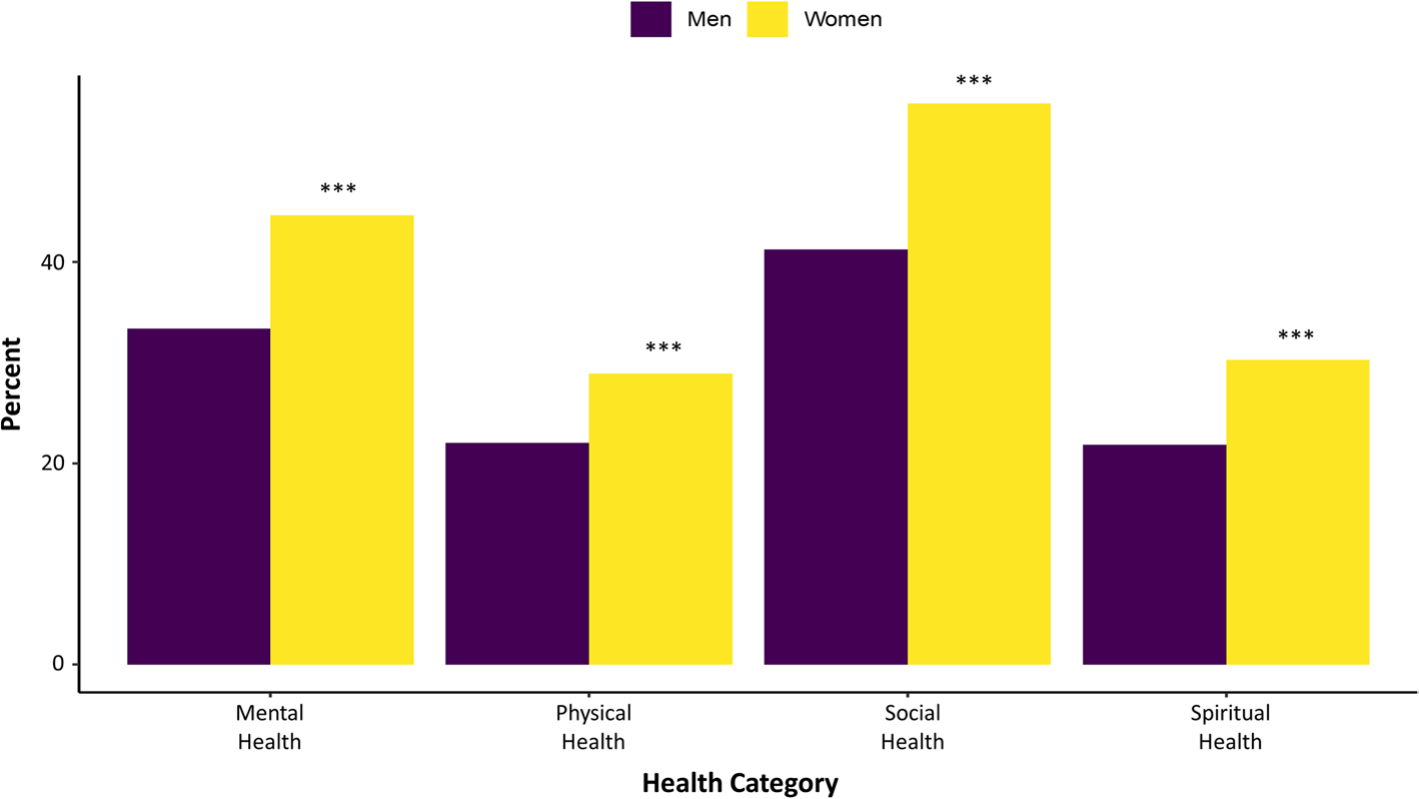
Percent of respondents showing worse health ratings compared to perceived health at the start of 2020 across four dimensions of health by gender. Legend: Odds Ratio significance indicates category differs from the reference group (18-29 year olds) at * *p* < 0.05, ** *p* < 0.01, and *** *p* < 0.001. Sample size: mental = 1973; physical = 1975; social = 1973; spiritual = 1875

**Fig. 2:**
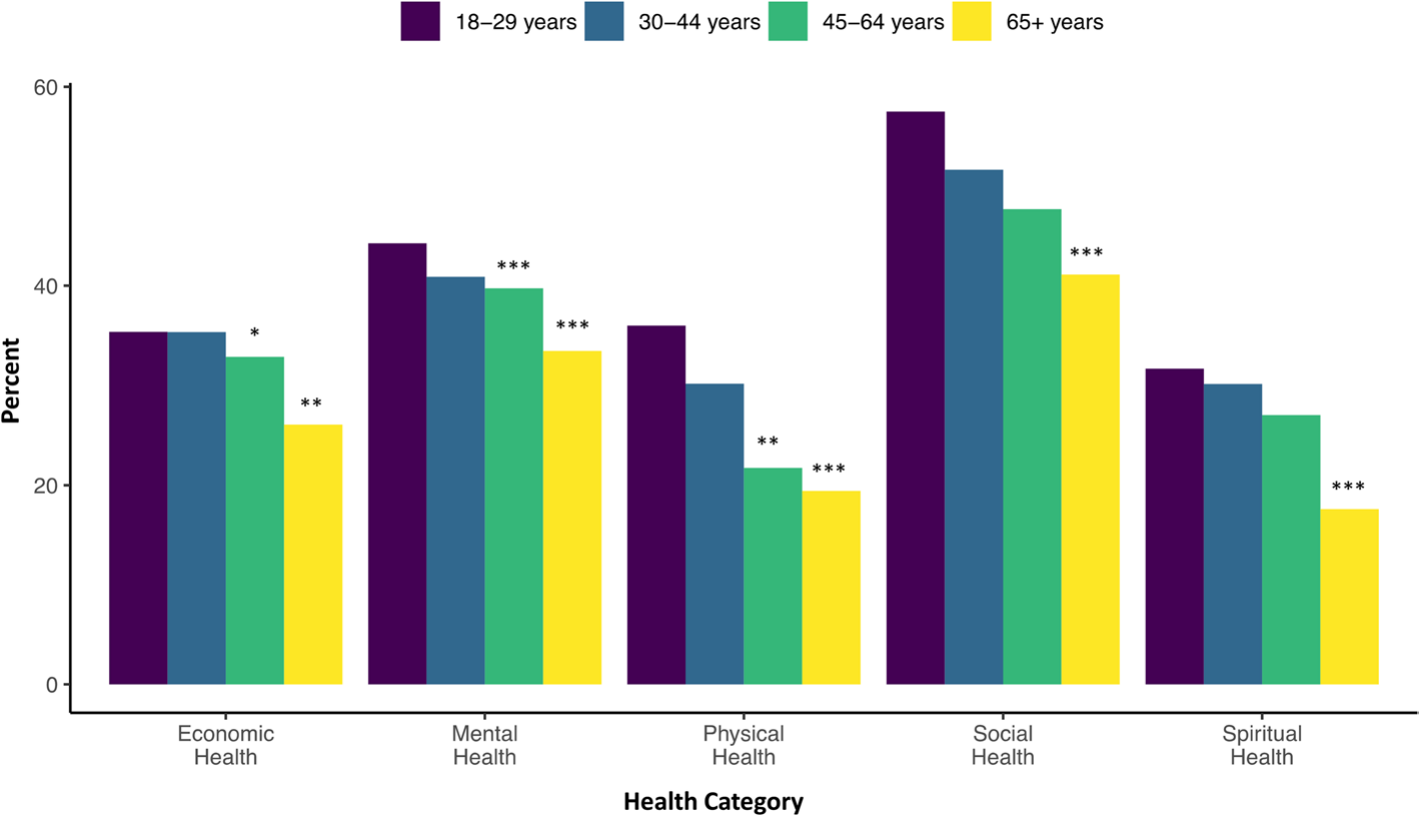
Percent of respondents showing worse health ratings compared to perceived health at the start of 2020 across five dimensions of health by age. Legend: Odds Ratio significance indicates category differs from the reference group (18-29 year olds) at * *p* < 0.05, ** *p* < 0.01, and *** *p* < 0.001. Sample size: economic = 1985; mental = 1988; physical = 1988; social = 1987; spiritual = 1888

**Fig. 3:**
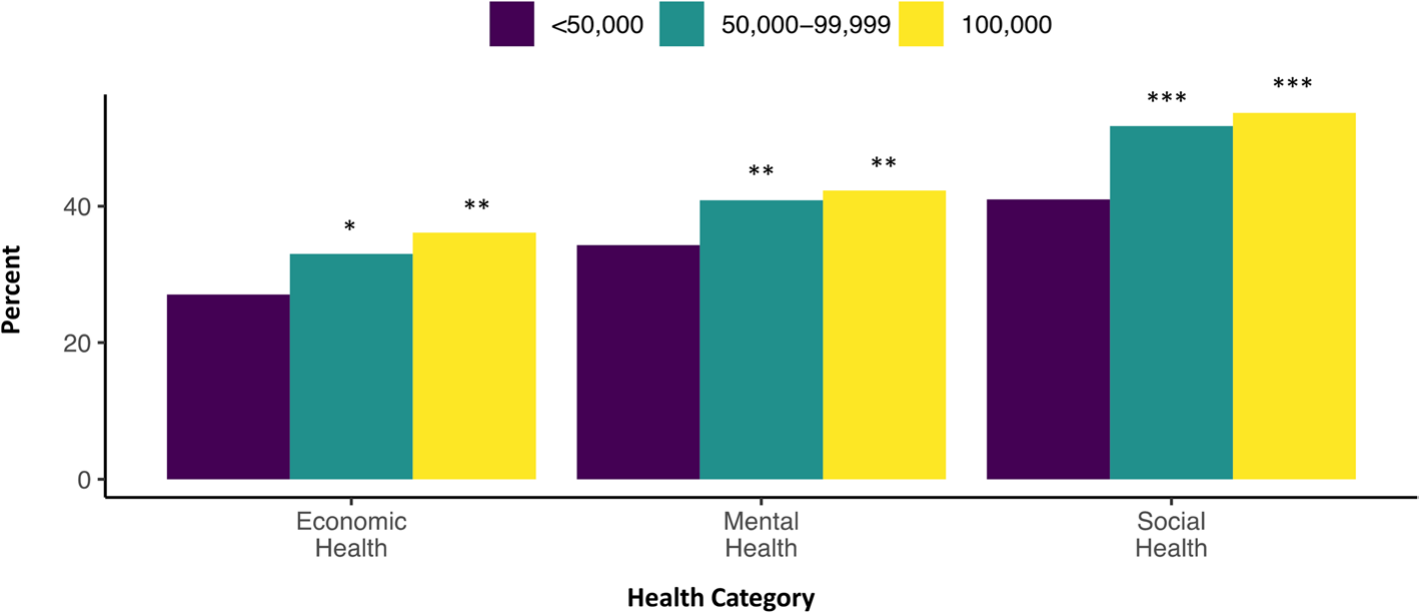
Percent of respondents showing worse health ratings compared to perceived health at the start of 2020 across three dimensions of health by income. Legend: Odds Ratio significance indicates category differs from the reference group (<$50,000) at * *p* < 0.05, ** *p* < 0.01, and *** *p* < 0.001. Sample size: economic = 1735; mental = 1742; social = 1741

There were differences in the proportions who reported COVID-19 as a ‘very serious’ problem based on gender and age. Women were more likely to report COVID-19 as a ‘very serious’ problem compared to men (OR 1.37, 95% CI 1.14, 1.64) and severity ratings proportionally increased with increasing age (see SFigure 1, Additional file 3). Relative to men, women were also more likely to ‘agree’ (OR 1.34, 95% 1.09, 1.64) or ‘strongly agree’ (OR 1.44, 95% CI 1.15, 1.81) that the pandemic has been stressful. This aligned with gender-based differences found in ratings of overall mental health as well as social and spiritual health at the time of the survey relative to the start of 2020, with women exhibiting worsening health compared to men (Fig. 1). There were age-group differences in all five domains of health (mental, physical, social, economic, and spiritual) (Fig. 2), and an income-group difference in social health (Fig. 3), but no differences based on education level. Respondents 65 and older were consistently less likely to report worse health ratings compared to the 18-29 age sub-group; the greatest spread between the oldest and youngest age groups was in overall physical health (Fig. 2). Respondents with $50 K to <$100 K (OR 1.55, 95% CI 1.23, 1.94) and $100 K+ (OR 1.67, 95% CI 1.31 2.13) household incomes were more likely to report worse social health compared to respondents with household incomes of <$50 K (Fig. 3).

**Fig. 4:**
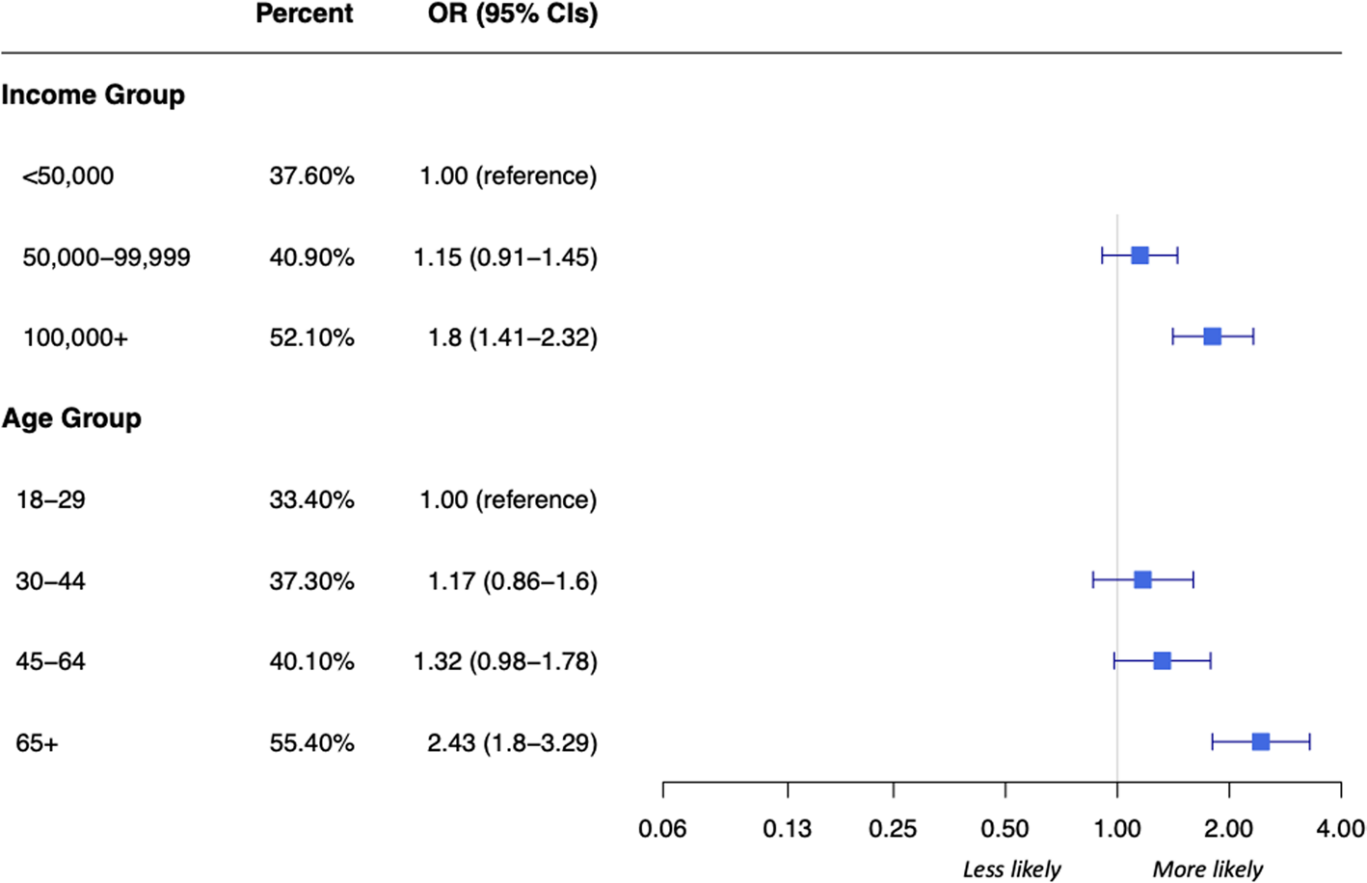
Percent of respondents by age group and income group that strongly agreed they would get a vaccine for COVID-19 once developed. Abbreviations: CIs, Confidence Intervals; OR, Odds Ratio. Legend: The x-axis utilizes a log scale. Sample size: Age, *n* = 1895; Income, *n* = 1076

Only an age group difference was found in the percentage of respondents that ‘always’ practiced physical distancing, which increased proportionally with increasing age (see SFigure 2, Additional file 3). Likewise, the duration that respondents believed they could sustain their current level of physical distancing was also related to age, with half of respondents 65 years and older believing they could sustain their current level longer than 6 months (or as long as needed) compared to one third of those age 18-29 (OR 2.14, 95% CI 1.59, 2.87) (see SFigure 3, Additional file 3). Similarly, when asked about the likelihood of getting vaccinated, respondents 65 years and older were 2.5 times more likely to ‘strongly agree’ that they intended to get vaccinated (OR 2.43, 95% CI 1.80, 3.29) (Fig. 4). We found a similar trend across income groups with respondents from $100 K+ households more likely to ‘strongly agree’ that they would get vaccinated compared with respondents from <$50 K households (OR 1.80, 95% CI 1.41, 2.32) (Fig. 4). Approximately three-quarters (*n* = 368; 74.9%) of $100 K+ household respondents ‘agreed’ or ‘strongly agreed’ that they would get vaccinated compared to just over half (*n* = 313; 55.5%) of <$50 K household respondents. The proportions of women and men were similar across all response options, and regression analyses did not result in significant differences between education sub-groups at 0.001.

### Access and Trust in Information Sources

Respondents accessed a mean of three (3.48 ± 0.06 (standard error)) sources from a predefined list of 19 sources for information about COVID-19, but respondents with high school education accessed fewer sources (2.59 ± 0.12) than the other education groups, as did respondents in $100 K+ households (3.82 ± 0.13) than those in <$50 K households (3.31 ± 0.11). Respondents age 18-29 accessed more information sources (mean = 3.92 ± 0.17) than those in older age groups, although they looked for information less frequently (see SFigure 4, Additional file 3). Respondents in age groups older than 18 to 29 years old were 1.7 (OR 1.68, 95% CI 1.17, 2.42) to 2.7 (OR 2.77, 95% CI 11.95-3.95) times more likely to report looking for information several times a day compared to those 18-29 years old (see SFigure 4, Additional file 3).

**Fig. 5:**
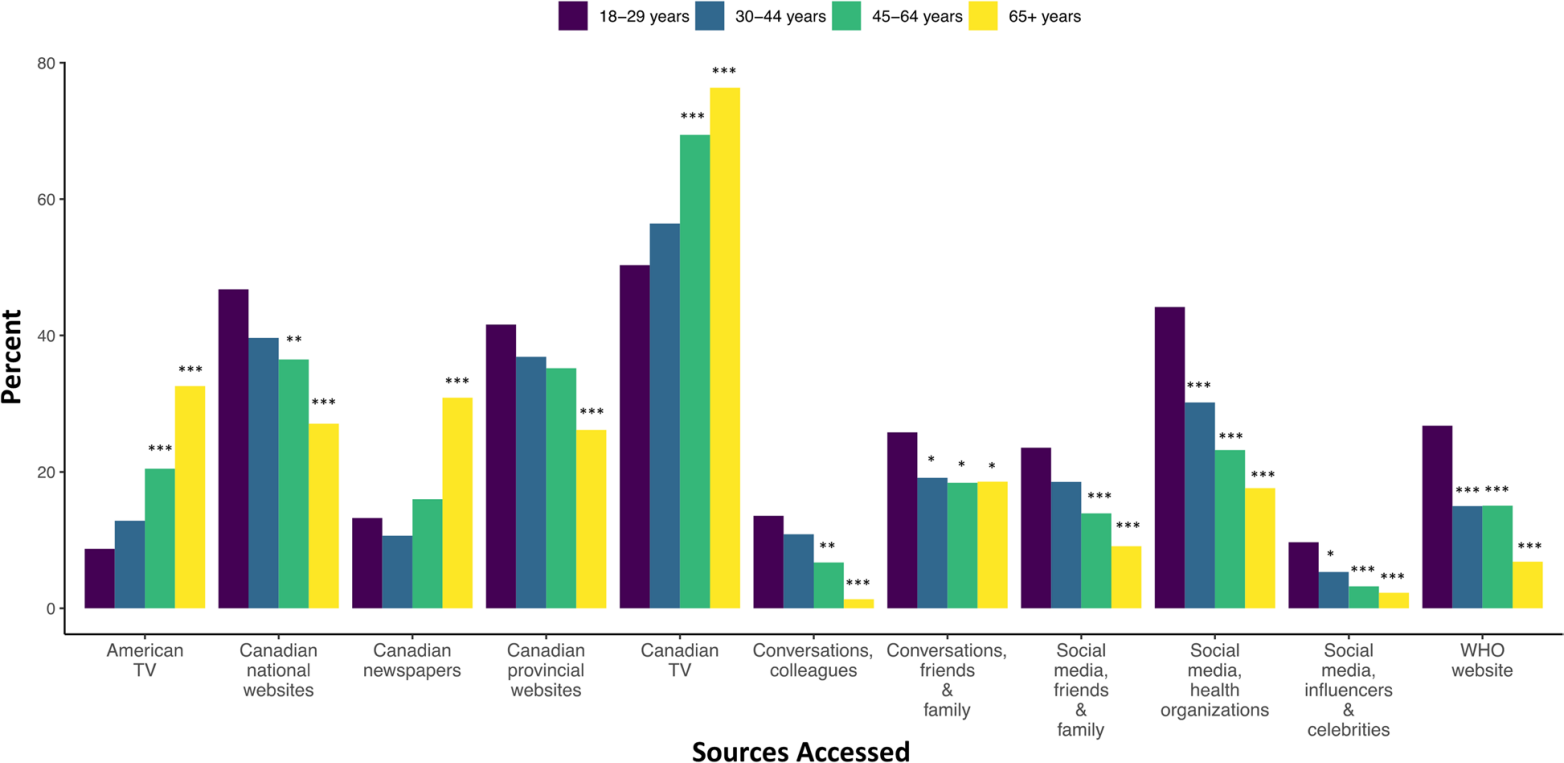
Information sources accessed for COVID-19 by age group (*n* = 1970). Abbreviations: TV, Television; WHO, The World Health Organization. Legend: Odds Ratio significance indicates category differs from the reference group (18-29 year olds) at * *p* < 0.05, ** *p* < 0.01, and *** *p* < 0.001

Canadian television news was the information source most commonly accessed for COVID-19 information by all sub-populations, but respondents age 45-64 (OR 2.25, 95% CI 1.69-3.00) and age ≥ 65 (OR 3.19, 95%CI 2.35-4.31) were two to three times more likely than respondents age 18–29 to access this source; the same trend was found in the odds of accessing American television news (Fig. 5). In contrast, respondents 65 years and older were also less likely to search for information through Canadian national (OR 0.42, 95% CI 0.31-0.57) or provincial (OR 0.50, 95% CI 0.37-0.67) websites compared with respondents age 18-29 (Fig. 5). The same pattern was found in accessing information through social media, particularly social media from health organizations, where 44.2% of 18-29 year old respondents reported searching for information; other age groups were roughly two to four times less likely to use this specific source (Fig. 5).

**Fig. 6:**
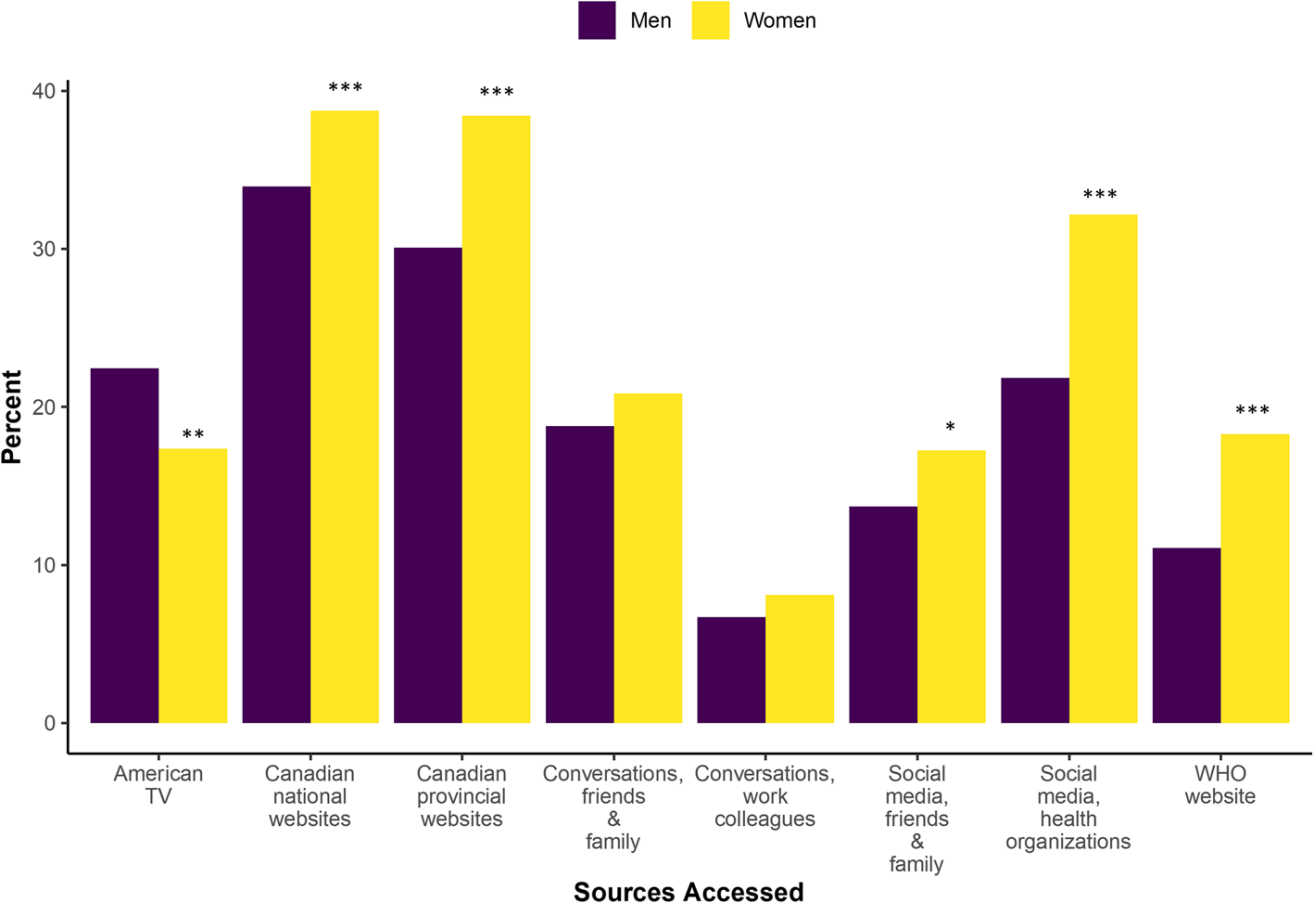
Information sources accessed by gender group (*n* = 1957). Abbreviations: TV, Television; WHO, The World Health Organization. Legend: Odds Ratio significance indicates category differs from the reference group (18-29 year olds) at * *p* < 0.05, ** *p* < 0.01, and *** *p* < 0.001

Women tended to access government and healthcare organization sources more than men. Specifically, women reported seeking information from Canadian national websites (OR 1.23, 95% CI 1.02, 1.48), Canadian provincial websites (OR 1.45, 95% CI 1.20, 1.75), the World Health Organization (WHO) website (OR 1.80, 95% CI 1.39, 2.35), and posts on social media from health organizations (OR 1.70, 95% CI 1.38, 2.09) (Fig. 6). Additionally, women were less likely than men to access American news sources for information (OR 0.68, 95% CI 0.55, 0.83) (Fig. 6).

**Fig. 7:**
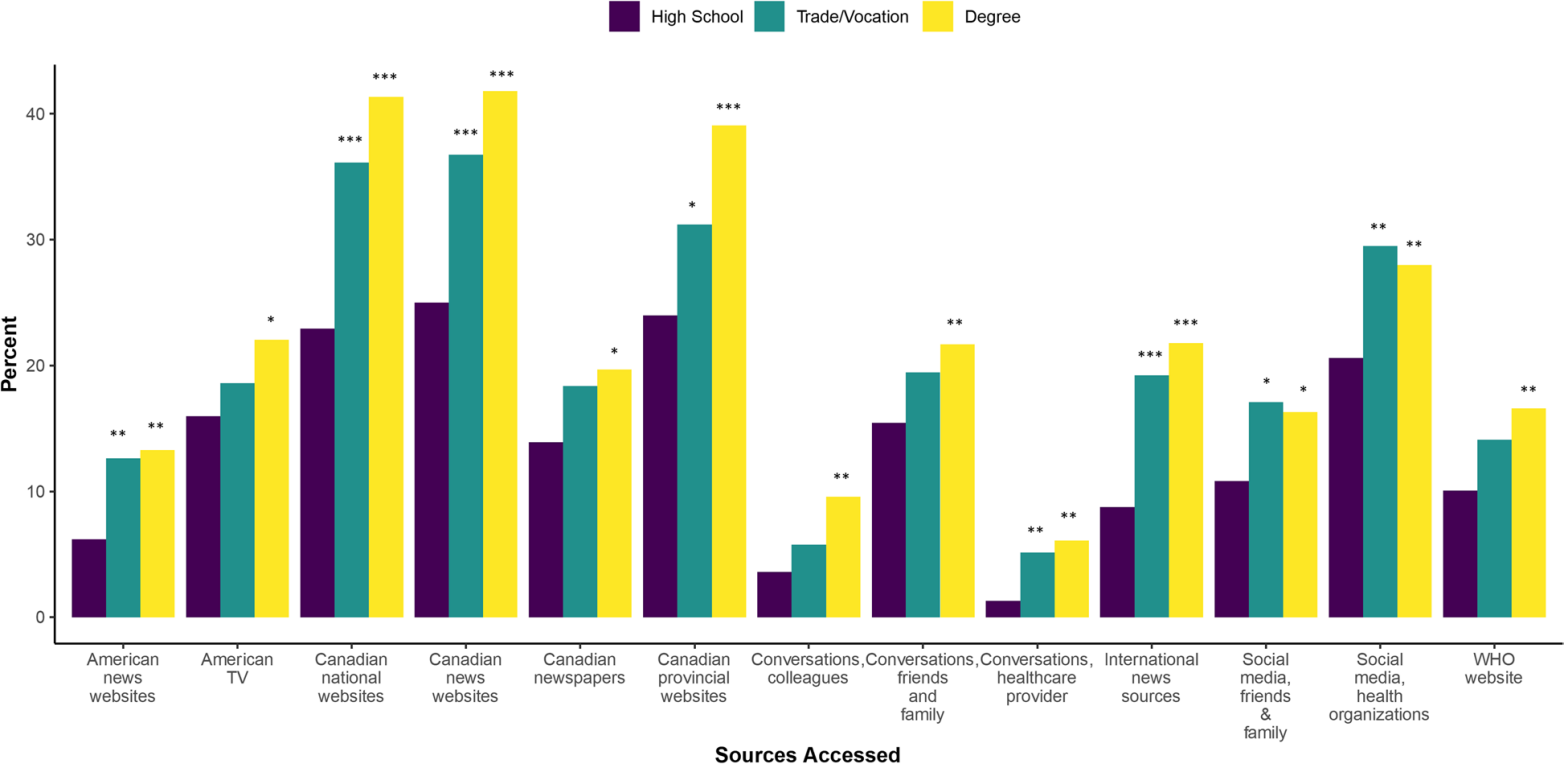
Information sources accessed by education group (*n* = 1954). Abbreviations: Degree, Post-secondary undergraduate, graduate, or professional degree; High school, high school diploma, Collège d’enseignement général et professionnel, or less; TV, Television; Trade/Vocational, Trade, Vocational certification and/or some university or college; WHO, The World Health Organization. Legend: Odds Ratio significance indicates category differs from the reference group (18-29 year olds) at * *p* < 0.05, ** *p* < 0.01, and *** *p* < 0.001

Compared to respondents with high school education, respondents with post-secondary education were more likely to access news and government sources for information on COVID-19, particularly through Canadian national government websites (OR (trade/some post-secondary) 1.90, 95% CI 1.40, 2.58; and OR (degree) 2.37, 95% CI 1.82, 3.10), Canadian news websites (OR (trade/some post-secondary) 1.73, 95% CI 1.28, 2.33, and OR (degree) 2.14, 95% CI 1.65, 2.77), and international news sources (OR (trade/some post-secondary) 2.49, 95% CI 1.63, 3.80, and OR (degree) 2.93, 95% CI 2.00, 4.29) (Fig. 7). Additionally, respondents with a post-secondary degree were more likely to report accessing Canadian provincial government websites (OR 2.02, 95% CI 1.55, 2.63), and American news sources (OR 1.73, 95% CI 1.30, 2.31) compared to respondents with high school education (Fig. 7).

**Fig. 8:**
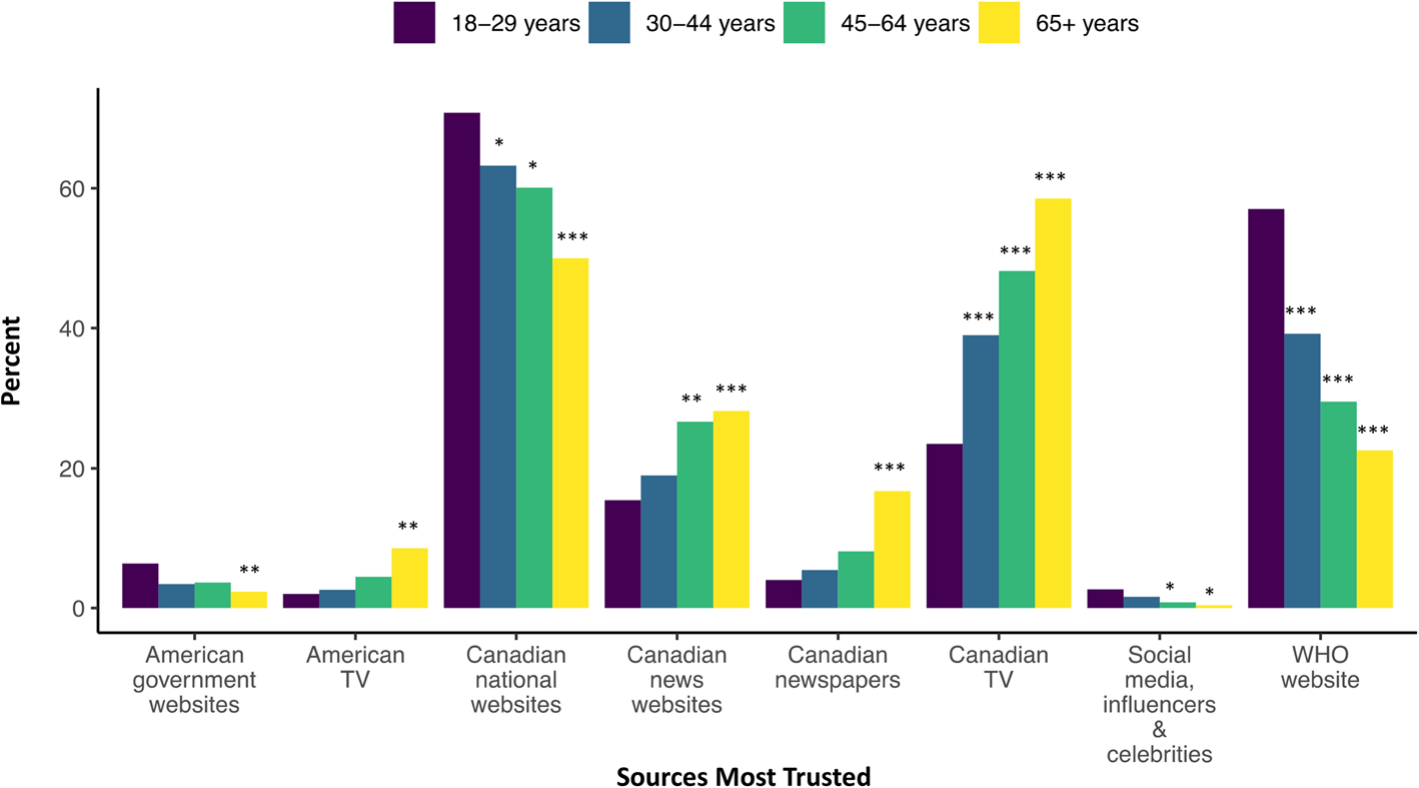
Information sources selected as most trustworthy by age group (*n* = 1911). Abbreviations: TV, Television; WHO, The World Health Organization. Legend: Odds Ratios significance indicates category differs from the reference group (18-29 year olds) at * *p* < 0.05, ** *p* < 0.01, and *** *p* < 0.001

The sources that respondents identified as most and least trustworthy paralleled the sources most and least accessed. The proportions who valued Canadian television as a trusted source increased with increasing age, with more respondents age ≥ 65 years trusting Canadian television compared to those age 18-29 (OR 2.96, 95% CI 2.02, 4.35) (Fig. 8). Respondents age 45-64 (OR 2.02, 95% CI 1.39, 2.92) and those age ≥ 65 (OR 2.18, 95% CI 1.50, 3.17) were more likely to select Canadian news websites as a trusted source compared to respondents age 18-29. Those age ≥ 65 were also more likely to identify Canadian newspapers as a trusted source compared to respondents age 18-29 (OR 4.94, 95% CI 2.59, 9.44), but less likely to trust Canadian national government websites (OR 0.41, 95% CI 0.30, 0.56). In comparison to the trend seen in age, the proportion of respondents who identified Canadian television as a trustworthy source decreased with increased education attainment (OR (degree) 0.66, 95% CI 0.52, 0.84); as well, respondents’ trust in Canadian national websites was highest for individuals with post-secondary education (65.2%) compared to individuals with high school education (51.9%) (OR 1.66, 95% CI 1.31, 2.12) (Fig. 8).

There were also differences in the reporting of trust in sources outside of Canada. For example, respondents age 65 and older were more likely to report American news websites (OR 2.01, 95% CI 1.35, 2.99) and American government websites (OR 2.84, 95% CI 1.98, 4.08) as least trustworthy compared to respondents age 18-29; respondents age 45-64 were also more likely to identify the latter as a least trusted source compared to the youngest age group (OR 1.96, 95% CI 1.36, 2.81) (see SFigure 5, Additional file 3). Identification of the WHO website as a trustworthy source differed by age and gender. Respondents age 30-44 (OR 0.49, 95% CI 01.36, 0.65), 45-64 (OR 0.49, 95% CI 01.36, 0.65) and ≥ 65 (OR 0.49, 95% CI 01.36, 0.65) were much less likely than respondents age 18-29 to identify the WHO website as a trusted source; and women were twice as likely to report trust in the WHO website compared to men (OR 1.93, 95% CI 1.59, 2.34). Women were also generally more likely to report social media posts from health organizations as most trustworthy compared to men (OR 1.33, 95% CI 1.04, 1.71) (see SFigure 6, Additional file 3), yet were more likely to report other posts on social media (posts from friends and family, and influencers or celebrities) as least trustworthy sources (see SFigure 7, Additional file 3). Trends in education subgroups were similar, in which respondents with a degree more frequently selected posts on social media from friends and family (OR 1.73, 95% CI 1.33, 2.25) or from influencers and celebrities (OR 1.78, 95% CI 1.40, 2.27) as least trustworthy sources compared to respondents with high school education. Respondents age 45-64 (OR 0.60, 95% CI 0.45, 0.80) and age ≥ 65 (OR 0.44, 95% CI 0.33, 0.60) were less likely to identify posts on social media from friends and family as a least trustworthy source compared to 18-29 year-old respondents.

COVID-19 information received from conversations with friends and family was less frequently identified as a least trustworthy source by respondents 45 years (OR 0.45, 95% CI 0.30, 0.67) and age ≥ 65 (OR 0.45, 95% CI 0.30, 0.68) compared to those aged 18-29 (see SFigure 5, Additional file 3) and by women compared to men (OR 1.52, 95% CI 1.15-2.02) (see SFigure 7, Additional file 3). Women were also more likely to select ‘information from acquaintances or individuals outside their circle’ as a least trustworthy source compared to men (OR 1.55, 95% CI 1.27, 1.89) (see SFigure 7, Additional file 3).

### Verifying information

About one-third of respondents across all demographic groups indicated to some degree that they found it hard to determine if an information source was trustworthy, but respondents with a post-secondary degree were more likely to disagree with this statement than respondents with a high school education (OR 2.11, 95% 1.45, 3.07). At the same time, 76.8% of all respondents reported at least one strategy that they used to verify or check information seen or heard. Respondents with post-secondary education were more likely than respondents with high school education to verify information through government or health authority sources (OR (trade/some post) 1.43, 95% CI 1.22, 2.37, and OR (degree) 1.65, 95% CI 1.31, 2.08), scientific articles (OR (trade/some post) 1.96, 95% CI 1.32, 2.87, and OR (degree) 1.93, 95% CI 1.36, 2.73), direct access of online news sources (OR (trade/some post) 1.70 95% CI 1.22, 2.37), and OR (degree) 1.75 95% 1.30, 2.34) and medical health professionals (OR (trade/some post) 1.94, 95% CI 1.32, 2.87, and OR (degree) 1.93, 95% CI 1.36, 2.73) (see SFigure 8, Additional file 3). Respondents age 65 and older were less likely to report going directly to an online news source (OR 0.54, 95% CI 0.39, 0.76) or government or health authority source (OR 0.52, 95% CI 0.39, 0.70) to verify information compared to respondents age 18-29, although government or health authority was still their most cited strategy. Respondents age 45-64 and age ≥ 65 were also less likely to report looking to scientific articles (OR (45-64) 0.51, 95% CI 0.38, 0.69, and OR (65+) 0.54, 95% CI 0.40, 0.73) and online search engines (OR (45-64) 0.52, 95% CI 0.39, 0.70, and OR (65+) 0.43, 95% CI 0.32, 0.58) compared to respondents age 18-29. Additionally, both of these age groups were more likely to report doing nothing to verify information compared to respondents age 18-29 (OR (45-64) 3.14, 95% CI 1.67, 5.91, and OR (65+) 4.60, 95% CI 2.47, 8.59) (see SFigure 9, Additional file 3). Results showed a gender-based difference in the reporting of two strategies to verify information. Women were more likely to report going directly to a government or health authority source than men (OR 1.56, 95% CI 1.31, 1.87) and less likely to use online search engines (OR 0.81, 95% CI 0.66, 0.98). Our analysis revealed no significant differences between household income groups in the information sources accessed or trusted.

### Information topics

Regardless of demographic grouping, infection rates and vaccine development or treatments were the leading topics searched for about COVID-19. However, respondents with a degree were more likely to search for information on infection rates compared with respondents with high school education (OR 1.55, 95% CI 1.21, 1.98), as were respondents 65 and older compared to respondents age 18-29 (OR 1.90, 95% CI 1.42, 2.53). Compared to respondents with high school education, respondents with a degree and those with a trade or some post-secondary education were also twice as likely to search for information on travel restrictions (OR (degree) 2.49, 95% CI 1.75, 3.55; OR (trade/some post) 2.00, 95%CI 1.35, 2.97). Age was associated with disproportionate searching for information on several other topics (see SFigure 10, Additional file 3). In comparison to respondents age 18-29, respondents age 65 and older were more likely to search for information on testing rates and procedures (OR 1.92, 95% CI 1.44, 2.56) and how they could personally prevent the spread of disease (OR 2.17, 95% CI 1.62, 2.89), while respondents age 30-44 were over twice as likely to search for information regarding their children’s education (OR 2.61, 95% CI 1.56, 4.36) (SFigure 10, Additional file 3). Comparatively, respondents in older age groups were less likely than respondents age 18-29 to search for information on accessing social services or resources (SFigure 10, Additional file 3).

## Discussion

This study analyzed the results of a pan-Canadian cross-sectional survey to examine sub-population differences in self-reported beliefs, prevention behaviours, and information acquisition related to the COVID-19 pandemic. We found important differences within the four demographic groups explored—binary gender, age, education, and income—with age effects most acutely evidenced in the data. Disproportionate responses between the oldest (≥65 years) and youngest (18 to 29 years) respondents were relatively consistent across all domains of inquiry (e.g., health, information-seeking). Our data also points to the disparate impact of COVID-19 on women and men’s health, as well as greater trust held by women in information disseminated by government and health authorities. Although there were fewer statistical differences between respondents based on household income and level of education, important differences within these demographics regarding intent to be vaccinated and in information sources accessed and trusted were evidenced. Since NPIs to control the spread of COVID-19 are intended to apply to all individuals, our findings highlight the importance of examining sociodemographic characteristics to contextualize and tailor messaging for the public [37].

The negative impact of COVID-19 on the self-reported mental health of women in our study relative to men is consistent with a growing body of research demonstrating the increased psychological burden women have carried throughout the pandemic [38, 39], including poorer quality of sleep, anxiety and depression, particularly while in self-isolation [40, 41]. However, sourcing the causes or correlates of pandemic-related mental health outcomes is complex. McElroy et al. [42] validated a Pandemic Anxiety Scale which revealed two distinct forms of pandemic-related anxiety: disease anxiety (e.g., catching, transmitting the virus) and consequences anxiety (e.g., impact on economics). Compared to men, they found that adult women scored higher on both forms of anxiety. Similarly, women in our study exhibited higher rates of concern about family members contracting the virus, concerns about insufficient personal protective equipment (PPE) in hospitals, and a general sense of helplessness due to the pandemic suggesting multi-faceted causes and implications.

Respondents in the oldest age group in our study perceived the severity of the pandemic more seriously than other age groups, but contrary to our gender analysis, they were less likely to report worse health ratings compared to younger respondents. This finding is supported by other polling data at the time of our survey which found that baby boomers felt relatively mentally and physically unaffected by the pandemic [43]. Likewise, adults 18-29 have reported higher levels of loneliness and psychological distress, compared to those aged 60-80 [44]. Greater struggles with unemployment [45] and loss of social networks during the pandemic may make it harder for younger adults to manage increased stress [46]. In our study, the highest proportion of respondents reporting worse social health was 18-29 year olds (60%); this group was also more likely to seek information on social programming and government support. Taking our gender and age-based findings together, women age 18-29 may be at greatest risk of negative consequences from the pandemic, a disparity that has been identified in other studies investigating social determinants of health during COVID-19 [44, 47] and previous outbreaks [48].

Several factors have been proposed to explain higher positive ratings of overall health by older adults including greater resilience to external stressors [49] and greater engagement in protective behaviours [48]. We found that individuals 65 and older were more consistently practicing physical distancing, felt more comfortable with sustaining these precautionary behaviours over a longer period of time, and were much more likely to indicate that they will get vaccinated than other age groups. These findings align with Bish and Michie’s [48] analysis of demographic and attitudinal determinants of protective behaviours during previous pandemics which demonstrated positive associations between older age and protective behaviors. Furthermore, psychological factors such as perceived risk to self and perceived efficacy of behaviors were noted to mediate the level of engagement in protective behaviors [48, 50, 51].

Data on access and use of information may provide some insights into understanding perceptions and behavioral intentions, particularly given the information-driven climate of the COVID-19 pandemic [28, 52–54]. Younger adults in our study more frequently used online technology including social media and websites to access information, while older respondents tended to rely more on television news and newspapers. Although social media is widely recognized as a source of misinformation and disinformation [55, 56], social media encompasses a broad range of online platforms and a broad range of users sharing content. Many individuals, as in our study, distinguish between social media from generally respected sources, such as the WHO, and those from unregulated sources (e.g., celebrities and influencers) which are more likely to propagate misleading or incorrect information [52, 56]. Furthermore, many respondents reported using reputable sources to help verify information that may be misleading or incorrect. However, whether this translates into informed decision-making and adherence to NPIs is unknown.

Ongoing trust in government and health institutions and the information they disseminate to the public may be the most critical factor to ensuring widespread “buy-in” of protective measures. In their study of coronavirus conspiracy beliefs, mistrust, and compliance with government guidelines in England, Freeman et al. [57] found that half their sample endorsed to some degree clearly false ideas about the pandemic, and that conspiracy beliefs were connected to mistrust in institutions and experts, among other correlates. Coupled with previous research substantiating links between distrust in government, limited access to health information, and vaccine hesitancy [58–60], particularly in marginalized communities who are generally less trusting of scientific organizations due to past systemic abuses [61, 62], concerns around sufficient vaccine uptake to contain SARS-CoV-2 are not unreasonable. Our data showed striking differences across demographic groups with younger adults and those with lower household income in particular less likely to agree that they would be vaccinated. Although the actions of public health authorities and policy makers bear much influence in the public’s level of trust, mass media (in all its forms) plays a critical role in the public’s evaluation of their legitimacy [59, 63–65]. This finding will be particularly relevant as vaccines are disseminated to the public.

### Limitations

We surveyed a large sample of adults living in Canada representative of the population by age, sex at birth, and geographical region. However, the survey was only available online and in English and French (Canada’s two official languages) limiting the opportunity to obtain the perspectives and experiences of non-users of the Internet (9% of Candians) [66] or those who did not speak English or French (1.8%) [11]. Moreover, we relied on a volunteer panel of potential participants (Ipsos’ iSay panel) which might have introduced bias, however, Ipsos applies rigorous processes (including ongoing monitoring and renewal) to ensure the source of panelists is representative of the population (including a mix of offline and online recruitment). Nevetheless, we were not able to ascertain how many panelists received our survey invitation and therefore not able to calculate a response rate. It is also important to note that the cross-sectional nature of this study limits the applicability of these findings over a longer period. However, the time frame of our survey provides a snapshot of a critical moment within the larger world event. When gathering data, we did not set sampling quotas for all demographics and consequently were not able to obtain sufficient variation across our sample to permit analysis by some important demographics such as ethnic origin and occupation. Nevertheless, we conducted a comprehensive exploration of the data by four key demographics applied to public health research. Future studies evaluating the perspectives, beliefs, and information acquisition across various ethnic groups would be important. Likewise, we collected global measures of self-reported health. Future studies should incorporate validated measures where feasible to detail associations between individual health and pandemic-related behaviors. Although these limitations highlight the need to expand the format and delivery of the survey as well as other variables that most reflect our diverse population, our findings can inform future studies about varied self-reported perceptions and behaviors during a pandemic.

## Conclusions

Our findings show that a “one-size fits all” approach to public health and science communication may be inadequate. Attention to population-based demographics, particularly age and gender, in knowledge translation strategies will help generate information relevant in format, tone, and medium to improve accessibility of information.

## Supplementary Information

**Additional file 1.** Public Perceptions Survey (English Version).

**Additional file 2:** **STable 1**. Respondent characteristics. **SFigure 1**. Pairwise comparisons of respondent age, gender, income, and education.

**Additional file 3: S1Figure.** Perception of COVID-19 pandemic severity by age group. **S2 Figure.** Self-reported frequency of physical distancing by age group. **S3 Figure.** Self-reported duration that current levels of physical distancing could be maintained by age group. **S4 Figure.** Self-reported frequency of information seeking by age group. **S5 Figure.** Information sources selected as LEAST trustworthy by age group. **S6 Figure.** Information sources selected as MOST trustworthy by gender group. **S7 Figure.** Information sources selected as LEAST trustworthy by gender group. **S8 Figure.** Self-reported strategies used to verify information by education level. **S9 Figure.** Self-reported strategies used to verify information by age group. **S10 Figure.** COVID-19 topics searched for information about by age groug.

## Data Availability

The datasets generated and/or analysed during the current study are not publicly available as we did not secure direct permission from the survey respondents to share the de-identified dataset with the general public. Requests for the deidentified data can be directed to the institutional research ethics boards overseeing the conduct of the study via the principal investigator, Dr. Jeanna Parsons Leigh (jparsonsleigh@dal.ca).

## Abbreviations

CDC: Center for Disease Control and Prevention
CI: Confidence Interval
COVID-19: Coronavirus disease of 2019
K: Thousand
NPI: Non-pharmaceutical Interventions
OR: Odds Ratios
SARS-CoV-2: Severe acute respiratory syndrome coronavirus 2
TV: Television
WHO: World Health Organization
