# Supplementary material for: Public perceptions during the first wave of the COVID-19 pandemic in Canada: a demographic analysis of self-reported beliefs, behaviors, and information acquisition
Source: BMC Public Health. 2022 Apr 9;22:699. doi: 10.1186/s12889-022-13058-3 (PMC8994420; doi:10.1186/s12889-022-13058-3)
Supplement: Supplementary file 1 — Additional file 1. Public Perceptions Survey (English Version). [file 12889_2022_13058_MOESM1_ESM.pdf]

Additional file 1.

Survey Development

To develop a 15-minute survey, we first drafted a comprehensive list of questions based on broad content areas reported in previously published survey research on pandemics[1] and in current COVID-19 public opinion polls.[2] We then created a list of questions in our key domains of interest—Beliefs, Knowledge, and Behaviors—and subsequently invited seven members of the research team (co-investigators, research assistants, and public partners) to provide feedback on question content as well as format, comprehensiveness, clarity, and flow, following the clinical sensibility assessment offered by Burns and colleagues.[3] We refined the questionnaire based on feedback.

Question types included 5-point unipolar scales (e.g., 1=not at all/poor, 5=extremely/excellent), 7-point bipolar agreement scales (1=strongly disagree to 7=strongly agree), single-response multiple choice, and multiple response multiple choice. We compared respondents' retrospective ratings of five domains of overall health (mental/emotional, physical, social, economic, spiritual) at the start of 2020 to ratings of their health status at the time of data collection, with differences categorized into 'worse', 'same', or 'better'. We provided respondents with definitions for self-isolation and social/physical distancing. Self-isolation was defined as *"separating yourself from others, including those within your home, with the purpose of preventing the spread of the virus (whether diagnosed or undiagnosed, with or without symptoms"* and social/physical distancing defined as *"limiting your time in spaces occupied by others, including reducing trips to visit others in person and reducing time spent in public spaces."*

We piloted the survey with a sample of 104 individual. No changes to the questionnaire were made. The questionnaire was optimized for compatibility across most devices (e.g., mobile phone, computer, tablet).

---

[1] Alqahtani AS, Rashid H, Basyouni MH, Alhawassi TM, BinDhim NF. Public response to MERS-CoV in the Middle East: iPhone survey in six countries. J Infect Public Health. 2017;10(5):534-40. Epub 2017/02/12. doi: 10.1016/j.jiph.2016.11.015.
Bener A, Al-Khal A. Knowledge, attitude and practice towards SARS. J R Soc Promot Health. 2004;124(4):167-70. Epub 2004/08/11. doi: 10.1177/146642400412400408..
Brug J, Aro AR, Oenema A, de Zwart O, Richardus JH, Bishop GD. SARS risk perception, knowledge, precautions, and information sources, the Netherlands. Emerg Infect Dis. 2004;10(8):1486-9. Epub 2004/10/22. doi: 10.3201/eid1008.040283.
de Zwart O, Veldhuijzen IK, Elam G, Aro AR, Abraham T, Bishop GD, et al. Perceived threat, risk perception, and efficacy beliefs related to SARS and other (emerging) infectious diseases: results of an international survey. Int J Behav Med. 2009;16(1):30-40. Epub 2009/01/07. doi: 10.1007/s12529-008-9008-2.
Burns KE, Duffett M, Kho ME, Meade MO, Adhikari NK, Sinuff T, et al. A guide for the design and conduct of self-administered surveys of clinicians. CMAJ. 2008;179(3):245-52. Epub 2008/07/30. doi: 10.1503/cmaj.080372.

[2] Canadian Research Insights Council. COVID-19 Public Opinion Research Hub 2019 [cited 2002 June 1, 2020]. Available from: https://www.canadianresearchinsightscouncil.ca/covid-19-resources-and-guidance/cric-covid-public-opinion-research-hub/.

[3] Burns KE, Duffett M, Kho ME, Meade MO, Adhikari NK, Sinuff T, et al. A guide for the design and conduct of self-administered surveys of clinicians. CMAJ. 2008;179(3):245-52. Epub 2008/07/30. doi: 10.1503/cmaj.080372. Clinical sensibility assessment questions (Likert 5-point rating scales).

- To what extent are the questions directed at important issues pertaining to the Canadian public's experiences of COVID-19?
- Are there important issues pertaining to public perceptions and experiences of COVID-19 that should be included in the questionnaire which have been omitted? Please identify any omissions
- To what extent are the response options provided simple and easily understood?
- To what extent are questions likely to elicit information pertaining to perceptions of and experiences with COVID-19?
- How many items are inappropriate or redundant? Please identify redudant or inappropriate items.
- How likely is the questionnaire to elicit a broad range of public perceptions of COVID-19 in the Canadian public?
- How long did it take you to complete the questionnaire?

Additional file 1.

Socio-Cultural Implications of COVID-19 Public Perceptions Survey

Dans quelle langue souhaitez-vous répondre à ce sondage? / In what language would you like to complete this survey?
O Français
O English

[After language selection, survey begins with implied consent in the appropriate language then on to Q1]

SCREENING QUESTIONS FOR QUOTA MANAGEMENT

YEAR / MONTH
Dropdown
What is your date of birth?

YEAR
_1918 1918
…
_2018 2018

MONTH
_1 January
_2 February
_3 March
_4 April
_5 May
_6 June
_7 July
_8 August
_9 September
_10 October
_11 November
_12 December

RESP_AGE
Single response

[PN: THANK AND TERMINATE IF UNDER 18]

S2. What was your assigned sex at birth (i.e., what does it say on your birth certificate)?
O Female
O Male
O Prefer not to answer

S3. What gender do you most identity with?
▢ Woman/girl
▢ Man/boy
▢ Non-binary
▢ Two-spirited

Additional file 1.

▢ Prefer to self-describe: __________
O Prefer not to answer

S4. What province or territory do you live in?
O British Columbia
O Alberta
O Saskatchewan
O Manitoba
O Ontario
O Quebec
O Newfoundland and Labrador
O Prince Edward Island
O New Brunswick
O Nova Scotia
O Yukon
O Northwest Territories
O Nunavut
O Prefer not to answer

MAIN QUESTIONNAIRE BODY

1. Are you aware of the current COVID-19 pandemic?
O Yes
O No
[IF Q.1="NO", GO TO 'READ SCREEN' AT THE BEGINNING OF THE DEMOGRAPHIC SECTION]

We would like to ask you some questions about the novel coronavirus that causes COVID-19. This virus is sometimes referred to as SARS-CoV-2 (severe acute respiratory syndrome coronavirus 2).
We are interested in your perspectives, experiences, and understanding of COVID-19.
Please answer all questions without checking the internet or other sources.

2. Have you had an illness that you believe was COVID-19 (currently or previously)?
O Yes, and I tested positive
O Yes, but I was not tested
O Yes, but I tested negative
O No
O Don't know
O Prefer not to answer

3. Do you know someone who has tested positive for COVID-19? Please select all that apply.
▢ Yes, family members living in the same house as me
▢ Yes, family members not living in the same house as me
▢ Yes, close friends
▢ Yes, colleagues or acquaintances
▢ No, I do not personally know anyone who has been diagnosed with COVID-19
O Prefer not to answer

4. How serious of a problem is COVID-19 in Canada currently?

O **O** **O** **O** **O** **O**

Additional file 1.

Not serious	Slightly serious	Somewhat serious	Moderately serious	Very serious	Prefer not to answer

5. **How serious of a problem is COVID-19 in Canada *compared to other countries in the world?***

O	O	O	O	O	O
Much less serious	Slightly less serious	About the same	Slightly more serious	Much more serious	Prefer not to answer

6. ***<u>At the start of 2020</u>*, how would you rate the following aspects of your overall health:**

[RANDOMIZE ITEMS]

	Poor	Fair	Good	Very good	Excellent	Prefer not to answer
Mental / emotional health	O	O	O	O	O	O
Physical health	O	O	O	O	O	O
Social health	O	O	O	O	O	O
Economic health	O	O	O	O	O	O
Spiritual health	O	O	O	O	O	O

7. ***<u>Currently</u>*, how would you rate the following aspects of your overall health:**

[ASK ITEMS IN Q.7 IN THE SAME ORDER AS RANDOMIZED IN Q.6]

	Poor	Fair	Good	Very good	Excellent	Prefer not to answer
Mental / emotional health	O	O	O	O	O	O
Physical health	O	O	O	O	O	O
Social health	O	O	O	O	O	O
Economic health	O	O	O	O	O	O
Spiritual health	O	O	O	O	O	O

8. **Please indicate how much you agree with each of the following statements:**

[RANDOMIZE ITEMS 8.1, 8.2 AND 8.3. ITEMS 8.4 AND 8.5 ARE ALWAYS ASKED LAST]

	Strongly Disagree	Disagr ee	Somewh at Disagree	Undeci ded	Somewh at Agree	Agree	Strong ly Agree	Prefer not to answer/ Don’t Know

Additional file 1.

**8.1.** The COVID-19 pandemic makes me feel helpless	O	O	O	O	O	O	O	O
**8.2.** The COVID-19 pandemic is very stressful	O	O	O	O	O	O	O	O
**8.3.** The *federal* government is NOT doing enough to support people affected by COVID-19	O	O	O	O	O	O	O	O
**8.4.** My *provincial* government is NOT doing enough to support people affected by COVID-19	O	O	O	O	O	O	O	O

9. **Please rate your degree of concern for each of the following statements.**

[ASK 9.1 ONLY IF Q.2="Yes, but I tested negative", "No" or "Don't know"]
[ALWAYS ASK 9.1 AND 9.2 FIRST]
[RANDOMIZE ITEMS 9.3, 9.4 AND 9.5]

	Not at all concerned	Slightly concerned	Somewhat concerned	Moderately concerned	Extremely concerned	Prefer not to answer/ Not Applicable
**9.1.** I will contract COVID-19	O	O	O	O	O	O
**9.2.** A family member will contract COVID-19	O	O	O	O	O	O
**9.3.** People will not be able to access the healthcare they need because of COVID-19 restrictions	O	O	O	O	O	O
**9.4.** There will not be enough protective equipment (e.g. masks) for hospital staff to stay safe	O	O	O	O	O	O

**9.5.** There will not be enough hospital equipment (e.g. beds) to care for patients with COVID-19	**O**	**O**	**O**	**O**	**O**	**O**

10. What concerns you the most about the COVID-19 pandemic?

[Open ended response to be coded]

O Prefer not to answer

11. How do you rate your general understanding of how the virus that causes COVID-19 is spread?

**O**	**O**	**O**	**O**	**O**	**O**
Poor	Fair	Good	Very Good	Excellent	Prefer not to answer

12. Please rate how much you agree with each of the following statements about the transmission of the virus that causes COVID-19:

[RANDOMIZE ITEMS]

	**Strongly disagree**	**Disagree**	**Somewhat disagree**	**Neutral**	**Somewhat agree**	**Agree**	**Strongly agree**	**Prefer not to answer/ Don't Know**
**12.1.** The virus can be spread through food and drink	**O**	**O**	**O**	**O**	**O**	**O**	**O**	**O**
**12.2.** People can be infected with COVID-19 but not show any symptoms	**O**	**O**	**O**	**O**	**O**	**O**	**O**	**O**
**12.3.** In order for the virus to spread, you need to have been in *close contact* with someone who has symptoms of COVID-19	**O**	**O**	**O**	**O**	**O**	**O**	**O**	**O**
**12.4.** Wearing a face mask when out in public can help prevent the virus from spreading	**O**	**O**	**O**	**O**	**O**	**O**	**O**	**O**
**12.5.** Nothing we do will stop the spread of the virus; we are all going to get COVID-19	**O**	**O**	**O**	**O**	**O**	**O**	**O**	**O**

eventually								
**12.6.** My province's testing procedures for COVID-19 (who is tested, number of tests) are sufficient	**O**	**O**	**O**	**O**	**O**	**O**	**O**	**O**

13. How often do you look for information about COVID-19?

**O**	**O**	**O**	**O**	**O**	**O**
Never	Once a week	Every couple of days	Once a day	Several times a day	Prefer not to answer

14. What sources have you gone to for information about COVID-19 over the last 2 weeks? Please select all sources that you accessed.

Governmental and public health website sources
- ☐ Canadian national websites
- ☐ Canadian provincial websites
- ☐ American government websites
- ☐ The World Health Organization's website

Television, print, or website sources
- ☐ Canadian television news
- ☐ American television news
- ☐ Canadian newspapers/magazines
- ☐ American newspapers/ magazine
- ☐ Canadian news websites
- ☐ American news websites
- ☐ International news sources including websites, broadcasts, and newspapers
- ☐ Other Websites

Social media sources
- ☐ Posts on social media from *health organizations and government*
- ☐ Posts on social media from *friends and family*
- ☐ Posts on social media from *influencers or celebrities*

Word-of-Mouth sources
- ☐ Conversations with friends and family
- ☐ Conversations with work colleagues
- ☐ Conversations with acquaintances or individuals outside my circle
- ☐ Conversations with a healthcare provider

☐ Other: **[PLEASE SPECIFY]**__________

O I have not gone to any source for information about COVID-19 during the past 2 weeks.
O None of the above
O Prefer not to answer

Additional file 1.

15. **What kind of information did you look for during the last 2 weeks related to the COVID-19 pandemic? Please select all that apply.**
    - ▢ Symptoms of COVID-19
    - ▢ Infection rates
    - ▢ Testing rates and procedures
    - ▢ Personal stories from others related to COVID-19
    - ▢ Personal stories from those who had/have COVID-19
    - ▢ Progress on development of a COVID-19 vaccine and potential vaccine safety
    - ▢ Treatments for COVID-19 currently in use or in development
    - ▢ How I can personally prevent spread of the disease
    - ▢ Access to social services or resources
    - ▢ Caring for a person who is at risk
    - ▢ Information on my children's education
    - ▢ Travel restrictions
    - ▢ Other: **[PLEASE SPECIFY]**__________

    O I did not look for any information related to COVID-19 the last two weeks
    O None of the above topics
    O Prefer not to answer

[PN: PLEASE USE DRAG AND DROP FOR Q.16]

16. **Please select and rank the 3 MOST trustworthy sources for COVID-19 information and the 3 LEAST trustworthy sources for COVID-19 information.**

Governmental and public health sources

- ▢ Canadian national websites
- ▢ Canadian provincial websites
- ▢ American government websites
- ▢ The World Health Organization's website

Television, print, or online sources

- ▢ Canadian television news
- ▢ American television news
- ▢ Canadian newspapers/magazines
- ▢ American newspapers/ magazine
- ▢ Canadian news websites
- ▢ American news websites
- ▢ International news sources including websites, broadcasts, and newspapers
- ▢ Other Websites

Social media sources

- ▢ Posts on social media from *health organizations and government*
- ▢ Posts on social media from *friends and family*
- ▢ Posts on social media from *influencers or celebrities*

Word-of-Mouth

- ▢ Conversations with friends and family
- ▢ Conversations with work colleagues
- ▢ Conversations with acquaintances or individuals outside my circle
- ▢ Conversations with a healthcare provider

▢ Other: **[PLEASE SPECIFY]__________**

O There are no trustworthy sources
O None of the above sources
O Prefer not to answer

17. How confident are you that *you* can identify incorrect or misleading information about COVID-19?

O	O	O	O	O	O
Not at all confident	Slightly confident	Somewhat confident	Moderately confident	Extremely confident	Prefer not to answer

18. How confident are you that *the average person* can identify incorrect or misleading information about COVID-19?

O	O	O	O	O	O
Not at all confident	Slightly confident	Somewhat confident	Moderately confident	Extremely confident	Prefer not to answer

19. Have you seen or heard incorrect or misleading information about COVID-19 in the last 2 weeks? Please select all topics that apply.

▢ Symptoms of COVID-19
▢ Infection rates
▢ Testing rates and procedures
▢ Personal stories from others related to COVID-19
▢ Personal stories from those who had/have COVID-19
▢ Progress on development of a COVID-19 vaccine and potential vaccine safety
▢ Treatments for COVID-19 currently in use or in development
▢ How I can personally prevent spread of the disease
▢ Access to social services or resources
▢ Caring for a person who is at risk
▢ Information on my children's education
▢ Travel restrictions
▢ Other: **[PLEASE SPECIFY]__________________________**
O I did not see or hear any incorrect or misleading information related to COVID-19 the last 2 weeks
O Prefer not to answer

[ASK Q.19.1 IF Q.19 IS NOT "I did not see or hear any incorrect or misleading information related to COVID-19 the last 2 weeks" or "Prefer not to answer"]

19.1. And where did you see or hear any incorrect or misleading information? Please select all that apply.

Governmental and public health website sources

▢ Canadian national websites
▢ Canadian provincial websites
▢ American government websites

- ▢ The World Health Organization's website

Television, print, or website sources

- ▢ Canadian television news
- ▢ American television news
- ▢ Canadian newspapers/magazines
- ▢ American newspapers/ magazine
- ▢ Canadian news websites
- ▢ American news websites
- ▢ International news sources including websites, broadcasts, and newspapers
- ▢ Other Websites

Social media sources

- ▢ Posts on social media from *health organizations and government*
- ▢ Posts on social media from *friends and family*
- ▢ Posts on social media from *influencers or celebrities*

Word-of-Mouth

- ▢ Conversations with friends and family
- ▢ Conversations with work colleagues
- ▢ Conversations with acquaintances or individuals outside my circle
- ▢ Conversations with a healthcare provider

▢ Other: **[PLEASE SPECIFY]**__________

O None of the above sources
O I don't remember
O Prefer not to answer

20. How do you verify or check the information that you see or hear? Please select all that apply.

- ▢ Use an online search engine (e.g., Google, Yahoo)
- ▢ Go directly to an online news source
- ▢ Go directly to a government or health authority source
- ▢ Look for scientific articles
- ▢ Ask a family member or friend
- ▢ Ask a medical health professional
- ▢ Ask on social media about the credibility of the information
- ▢ Use Snopes, or another fact-checking service
- ▢ I do not use any of the above strategies
- ▢ Other: **[PLEASE SPECIFY]**__________

O Nothing, I just choose to not believe it
O Nothing, I just choose to believe it
O Prefer not to answer

21. Please indicate how much you agree with each of the following statements:

[RANDOMIZE ITEMS]

	**Strongly**	**Disagree**	**Some-what**	**Neutral**	**Some-what**	**Agree**	**Strongly**	**Prefer not to**

Additional file 1.

	disagre e		Disagr ee		Agree		agree	answe r/ Don't know
**21.1.** I am able to find the kind of information I want about COVID-19	**O**	**O**	**O**	**O**	**O**	**O**	**O**	**O**
**21.2.** I find it hard to determine if an information source is trustworthy or not.	**O**	**O**	**O**	**O**	**O**	**O**	**O**	**O**

22. Are you currently in self-isolation?

Self-isolation is when you are separating yourself from others, including those within your home, with the purpose of preventing the spread of the virus (whether diagnosis or undiagnosed, with or without symptoms). You do not leave the house, go to work, or get groceries or access other essential services

O Yes, I am currently in self-isolation
O No, I am not currently in self-isolation
O Prefer not to answer

[ASK Q.23 IF Q.22 = "No, I am not currently in self-isolation" or "Prefer not to answer"]

23. How often are you practicing social or physical distancing?

Social or physical distancing refers to limiting your time in spaces occupied by others, including reducing trips to visit others in person and reducing time spent in public spaces.
The World Health Organization has recommended that 'physical distancing' is the most appropriate term to use to describe these social measures. As a result, for the rest of the survey, we will use physical distancing.

**O**	**O**	**O**	**O**	**O**	**O**
Not at all	Rarely	Sometimes	Often	Always	Prefer not to answer

[ASK Q.24 IF Q.22 = "Yes, I am currently in self-isolation"]

24. Why have you been practicing self-isolation? Please select up to 3 of your top reasons.

❑ To protect myself
❑ To protect other people I live with
❑ To protect other people that I live with who are vulnerable/high risk
❑ To protect other family members or friends that I do not live with
❑ To protect other members of the general public
❑ To help decrease the burden on the healthcare system
❑ To avoid possibly receiving a fine for not following guidelines.
❑ Other: **[PLEASE SPECIFY]**__________
O Prefer not to answer

[ASK Q.25 IF Q.23 = "Sometimes", "Often" or "Always"]

Additional file 1.

25. **Why have you been practicing physical distancing? Please select up to 3 of your top reasons.**
    - ▢ To protect myself
    - ▢ To protect other people I live with
    - ▢ To protect other people that I live with who are vulnerable/high risk
    - ▢ To protect other family members or friends that I do not live with
    - ▢ To protect other members of the general public
    - ▢ To help decrease the burden on the healthcare system
    - ▢ To avoid possibly receiving a fine for not following guidelines.
    - ▢ Other: **[PLEASE SPECIFY]__________**
    - **O** Prefer not to answer

[ASK Q.26 IF Q.23 = "Not at all" or "Rarely"]

26. **Why are you not regularly practicing physical distancing? Please select all that apply.**
    - ▢ I am healthy
    - ▢ I am not concerned about getting the virus that causes COVID-19
    - ▢ I think the situation is not as bad as people are saying it is
    - ▢ It is important for me to continue to visit friends and/or family member during the pandemic
    - ▢ My work makes it very difficult to practice distancing
    - ▢ Everyone will get the virus that causes COVID-19 eventually, so it does not matter.
    - ▢ Other: **[PLEASE SPECIFY]__________**
    - **O** Prefer not to answer

[ASK Q.27 IF Q.24 = "To protect other people that I live with who are vulnerable" OR IF Q.25 = "To protect other people that I live with who are vulnerable"]

27. **You indicated that you are practicing self-isolation or social or physical distancing to protect someone who is vulnerable/high-risk. Please describe the vulnerable people you are protecting by practicing self-isolation or physical distancing, and describe the reason they are vulnerable.**

    [Open ended response to be coded]

    O Prefer not to answer

[If Q.22 = "Yes, I am currently in self-isolation", include READ SCREEN below]
[READ SCREEN]

Social or physical distancing refers to limiting your time in spaces occupied by others, including reducing trips to visit others in person and reducing time spent in public spaces. The World Health Organization has recommended that 'physical distancing' is the most appropriate term to use to describe these social measures. As a result, for the rest of the survey, we will use physical distancing.

[ASK ALL]

28. **Which age group do you think is practicing physical distancing <u>most consistently</u>?**
    - **O** Teenagers
    - **O** Young adults
    - **O** Middle-aged adults
    - **O** Seniors
    - **O** Don't know
    - **O** Prefer not to answer

29. **Which age group do you think is practicing physical distancing <u>least consistently</u>?**
    - **O** Teenagers
    - **O** Young adults
    - **O** Middle-aged adults

O Seniors
O Don't know
O Prefer not to answer

30. How much longer do you believe you can reasonably sustain your *current* level of physical distancing?

O Not any longer
O 2 more weeks
O 1 more month
O 2 more months
O 3 more months
O 6 more months
O Longer than six months (as long as it is needed)
O Don't know
O Prefer not to answer

31. Please indicate how much you agree with each of the following statements:

[RANDOMIZE ITEMS]

	**Strongly disagree**	**Disagree**	**Some-what disagree**	**Neutral**	**Some-what agree**	**Agree**	**Strongly agree**	**Prefer not to answer / Don't know**
**31.1.** With changes to my behaviour, I am doing a good job at preventing the spread of the virus that causes COVID-19	**O**	**O**	**O**	**O**	**O**	**O**	**O**	**O**
**31.2.** I am doing a better job than most Canadians changing my behaviour to prevent the spread of the virus that causes COVID-19	**O**	**O**	**O**	**O**	**O**	**O**	**O**	**O**
**31.3.** I will get vaccinated for the virus when it is developed	**O**	**O**	**O**	**O**	**O**	**O**	**O**	**O**

You have reached the end of this portion of the survey. Thank you for your responses!

Additional file 1.

Demographics

[READ SCREENa IF Q1=NO]
There is a pandemic of respiratory disease caused by the novel coronavirus, also called COVID-19. This virus was first discovered in Wuhan, Hubei province in China and has since spread across the world. There are hundreds of thousands of confirmed cases and many deaths related to COVID-19, including in Canada. Please visit the Government of Canada COVID-19 Website for information.

[READ SCREEN FOR ALL]
We would now like to ask you some demographic questions to help categorize the responses. As a reminder, your answers will only be used in grouped data analysis – we will not be able to identify you.

D1. What is your current marital status?
O Single, never married
O In a relationship, but not living together
O Living with partner
O Married
O Separated or divorced (in process or finalized)
O Widowed
O Prefer not to answer

D2. What is the size of the town or city you live in?
O An unincorporated area (e.g. township, village – up to 1,000 people)
O Small town or village (up to 5,000 people)
O Small city (up to 10,000 people)
O Medium sized city (over 10,000 people up to 100,000 people)
O Large city (over 100,000 people up to 1,000,000 people)
O Large metropolitan area (over 1,000,000 people)
O Don’t know
O Prefer not to answer

D3. How many people live in your household, including yourself?
(Please include all other family members and / or roommates living in the same house)
___ person / people [verification: integer]
O Prefer not to answer

D4. How many children (under the age of 18) live in your household? Please do not include children who are away at school. *(If you do not have any children in a specific age range, please enter ‘0’)*
O I do not have any children under the age of 18 years living at home
___ infants(s) (<=1 years) [verification: integer]
___ toddler(s) (1- 2 years) [verification: integer]
___ child(ren) (3 -12 years) [verification: integer]
___ teenager(s) (13-17 years) [verification: integer]
O Prefer not to answer

D5. What is your current employment status? Please select all that apply.
☐ Employed (working full-time hours)
☐ Employed (working part-time/casual hours)
☐ Self-employed (working full-time hours)
☐ Self-employed (working part-time hours)
☐ Retired
☐ Student (full or part-time)

▢ Full time parent or homemaker
▢ Military (full or part-time)
▢ Currently unemployed or unable to work for any reason (including laid off)
O Prefer not to answer

[ASK QD6 IF D5 = "Currently unemployed or unable to work for any reason (including laid off)"]
D6. **Is your current unemployment <u>a direct result</u> of the COVID-19 pandemic (e.g. laid off by a company, ordered to discontinue service from the government)?**
O Yes
O No
O Unsure
O Prefer not to answer

[ASK D7 IF D5 = "Employed (working full-time hours)", "Employed (working part-time/casual hours", "Self-employed (working full-time hours)", "Self-employed (working part-time hours)", "Military (full or part-time)" or "Currently unemployed or unable to work for any reason (including laid off)"]
D7. What type of industry do you work in/did you most recently work in? Examples are included. Please select the most appropriate category.
O Hospital healthcare professional (physician, nurse, respiratory therapist, medical lab assistant, etc.)
O Hospital support staff (environment and cleaning, food service, etc.)
O Community healthcare professional (physical therapist, psychologist, dietician, etc.)
O Dental industry (dentist, hygienist, assistant, etc.)
O First responder (police, fire, paramedic services)
O Restaurant, bar, or nightclub industry
O Energy industry (oil, gas, solar, hydro, etc.)
O Service industry (grocery stores, hardware stores, liquor stores, etc.)
O Agricultural and natural resource sector (farming, logging, mining, etc.)
O Construction industry, other material building suppliers, and related trades (residential or commercial, including a contractor or sub-contractor)
O Government / public service sector (federal, provincial, or municipal)
O Entertainment (music, arts, theatre, photography, etc.)
O Primary or secondary education (public or private)
O Post-secondary education, academia, and research (including support staff)
O Industry sponsor research (e.g. private companies)
O Other: **[PLEASE SPECIFY]** ________________
O Prefer not to answer

D8. Has the government identified your occupation as an essential service?
O Yes
O No
O Unsure
O Prefer not to answer

D9. How many incomes contribute to your overall household income?
_____ income(s) [verification: integer]
O Prefer not to answer

D10. What was your total overall household income in 2019 before taxes?
O $0
O $1 to $9 999
O $10 000 to $24 999
O $25 000 to $49 999

O $50 000 to $74 999
O $75 000 to $99 999
O $100 000 to $149 999
O $150 000 to $249 999
O $250 000 or more
O Don't know
O Prefer not to answer

D11. As you know, we all live in Canada, but we come from many different ethnic backgrounds. What is your **main** ethnic background? Please select up to two responses.

O ***Canadian/French Canadian***
O ***Caucasian/White***
O ***British*** (English/Scottish/Welsh/Irish)
O ***Western European*** (from Austria, Belgium, France, Germany, Netherlands, or other)
O ***Southern or Eastern European*** (from Greece, Italy, Portugal, Spain, Bosnia, Croatia, Serbia, Czech Republic, Hungary, Poland, Slovakia, Ukraine, former Soviet Republics, or other)
O ***South Asian*** (Punjabi, Indian, Tamil, Sri Lankan, Pakistani, Bangladeshi, Nepalese)
O ***East or Southeast Asian*** (from China, Hong Kong, Japan, North or South Korea, Indonesia, Malaysia, Philippines, Singapore, Thailand, Vietnam or other)
O ***West Asian or Middle Eastern*** (from Afghanistan, Iran, Iraq, Israel, Lebanon, Saudi Arabia, Syria, Turkey or other)
O ***African***
O ***Central/South American or Caribbean*** (from Argentina, Brazil, Columbia, El Salvador, Guatemala, Mexico, Venezuela, Barbados, Jamaica, or other)
O ***Aboriginal/First Nations/Métis***
O Other (Specify)
O Prefer not to answer

D12. Which of the following best describes your religious identity?

[SINGLE PUNCH]
O Roman Catholic
O Protestant or other Christian
O Muslim
O Jewish
O Hindu
O Sikh
O Other **[SPECIFY]**___________________
O No Religious Identity
O Don't know/Prefer not to answer

D13. **Thinking of how you feel right now, if a FEDERAL election were held tomorrow, which of the following parties' candidates would you, yourself, be most likely to support?***Please select one response only.*

[RANDOMIZE CODES 1-5]
O The Conservative Party
O The Liberal Party
O The New Democratic Party (NDP)
O **[QUÉBEC ONLY]** The Bloc Québécois (BQ)
O The Green Party
O **[SHOW FOURTH TO LAST]** Some other independent party

O [SHOW THIRD TO LAST] Would not vote/None/Would spoil ballot)
O [SHOW SECOND TO LAST] Don’t know/not sure
O [SHOW LAST] Prefer not to answer

[IF DON'T KNOW OR NOT SURE IN QD13, ASK QD13b, ELSE SKIP TO QD14]
D13b. Well, which party would you say you would lean towards? *Please select one response only.*

[SAME ORDER AS QD13]
O The Conservative Party
O The Liberal Party
O The New Democratic Party (NDP)
O The Green Party
O [QUÉBEC ONLY] The Bloc Québécois (BQ)
O [SHOW THIRD TO LAST] Some other independent party
O [SHOW SECOND TO LAST] Don’t know/not sure
O [SHOW LAST] Prefer not to answer

D14. What is the highest level of education have you completed?
O Less than a high school diploma
O High school diploma
O CEGEP
O Vocational college
O Trade certification
O Some college (no degree)
O College degree
O Some university (no degree)
O Undergraduate degree (Bachelor’s)
O Graduate degree (Masters or Doctorate)
O Professional degree (MD, JD, DDS, etc.)
O Prefer not to answer

D15. What kind of residence do you live in?
O Detached home
O Semi-detached home (e.g. duplex, townhouse)
O Apartment or condominium
O School residence or dormitories
O Assisted living or care home
O Other communal housing (e.g. transition house, shelter)
O Other: **[PLEASE SPECIFY]**__________
O Prefer not to answer

[ASK QD16 IF QD15 = “Apartment or condominium”, “School residence or dormitories”, “Assisted living or care home”, or “Other communal housing (e.g. transition house, shelter”]
D16. Have any specific regulations or policies in response to COVID-19 been implemented in your building (e.g. supplemental cleaning procedures, elevator occupancy limits, etc.)?
O Yes
O No
O Unsure
O Prefer not to answer

D17. Are you currently diagnosed with any of the following health conditions? Please select all that apply.
Please do not select any conditions you may have been previously diagnosed but no longer have.

▢ Autoimmune disease (e.g. lupus, psoriasis, rheumatoid arthritis, Crohn's disease, etc.)
▢ Cancer (currently in treatment)
▢ Chronic lung disease (e.g. COPD, asthma, etc.)
▢ Dementia
▢ Diabetes (Type 1 or Type 2)
▢ Cardiovascular disease (e.g. arrhythmias, coronary artery diseases, hypertension, valvular diseases, etc., including congenital diseases)
▢ Mental health (e.g. anxiety, depression, post-traumatic stress disorder, substance use disorder, etc.)
▢ Obesity
▢ Other chronic diseases (e.g. high cholesterol, kidney (renal) disease, osteoarthritis, neuromuscular, etc.)
▢ Other: **[PLEASE SPECIFY]**__________
O I am not currently diagnosed with any of these health conditions
O Prefer not to answer

Thank you for your participation.

Your participation provides important insights that will be used to help improve the COVID-19 pandemic response. For more information about COVID-19 please visit please visit: www.Canada.ca/covid-19 .

If you have questions, please contact C3ResNetwork@ucalgary.ca.

SUBMIT
