# Supplementary material for: Public perceptions during the first wave of the COVID-19 pandemic in Canada: a demographic analysis of self-reported beliefs, behaviors, and information acquisition
Source: BMC Public Health. 2022 Apr 9;22:699. doi: 10.1186/s12889-022-13058-3 (PMC8994420; doi:10.1186/s12889-022-13058-3)
Supplement: Supplementary file 2 — Additional file 2: STable 1. Respondent characteristics. SFigure 1. Pairwise comparisons of respondent age, gender, income, and education. [file 12889_2022_13058_MOESM2_ESM.pdf]

Additional File 2.

Respondent Characteristics

STable 1. Respondent characteristics (total sample size=1,996) 2
SFigure 1. Pairwise Comparisons of Respondent Age, Gender, Income, and Education (n=1,996) 5

Additional File 2.

STable 1: Respondent characteristics (total sample size=1,996)

Participant Characteristics		Number (%)[1]
**Gender**	(n=1,988)	
Woman/girl		1080 (54.3)
Man/boy		899 (45.2)
Other, self-described		9 (0.5)
**Age (in years)**	(n=1,996)	
Mean (SD)		50 (34-66)
18-29		303 (15.2)
30-44		505 (25.3)
45-64		637 (31.9)
65+		551 (27.6)
**Region**[2]	(n=1,996)	
British Columbia		271 (13.6)
Alberta		224 (11.2)
Manitoba/Saskatchewan		130 (6.5)
Ontario		767 (38.4)
Québec		468 (23.4)
Maritimes[3]		136 (6.8)
**City Size**	(n=1,965)	
Small town or city (up to 10,000 people)		389 (19.8)
Medium size city (>10,000 to <100,000)		466 (23.7)
Large city (>100,000 – 1,000,000)		622 (31.7)
Large metropolitan area (>1,000,000)		488 (24.8)
**Ethnic Origins**[4]	(n=1,967)	
Canadian/French Canadian		709 (36.0)
European		606 (30.8)
East or Southeast Asian		101 (5.1)
South Asian		54 (2.7)
Other[5]		139 (7.1)
Caucasian/White		914 (46.5)
**Religious Identity**	(n=1,935)	
Catholic/Protestant/Christian		1091 (54.7)
Muslium		37 (1.9)
Jewish		42 (2.1)
Other[6]		90 (8.2)
Non-religious		675 (33.8)
**Marital Status**	(n=1,985)	
Single, never married		493 (24.8)
Partnered[7]		1,214 (61.2)
Separated/divorced/widowed		277 (14.0)
**Highest Education**	(n=1,975)	
High school equivalent, or less		396 (20.1)
Trade or technical college; some college/university		475 (24.1)
College/University/Postgraduate degree		1,104 (55.9)

Additional File 2.

**Individuals in Household**		
Median (IQR)		2 (2-3)
Do not have children living in household	(n=1,995)	1583 (81.0)
Have children living in household		412 (20.6)
Infant(s) (<=1 year)		43 (10.4)
Toddler(s) (1-2 years)		44 (10.7)
Child(ren) (3-12 years)		245 (59.5)
Teenager(s) (13-17 years)		178 (43.2)
**Total Household Income**	(n=1,741)	
0$ - $49,999		600 (34.5)
$50,000 - $99,999		658 (37.8)
$100,000 or more		483 (27.8)
**Type of Residence**	(n=1,975)	
Detached home		1084 (54.9)
Semi-detached home (e.g., duplex, townhouse)		295 (14.8)
Apartment or condominium		564 (28.6)
Shared/communal housing/Other		32 (1.6)
**Federal Political Party Alignment**	(n=1,912)	
The Conservative Party		427 (22.3)
The Liberal Party		626 (32.6)
The New Democratic Party		222 (11.6)
Other political parties		182 (9.5)
Would not vote/would spoil ballot/not sure		459 (24.0)
**Employment**	(n=1,968)	
Employed (working full-time hours)		777 (39.5)
Employed (working part-time/casual hours)		201 (10.2)
Retired		567 (28.8)
Not employed (student/homemaker/unemployed)		423 (21.5)
Unemployed as a result of COVID-19	(n=282)	
Yes		143 (50.7)
Essential worker status	(n=1,996)	
Yes		550 (27.6)
Employment Sector	(n= 1,171)	
Hospital healthcare professional		43 (3.4)
Hospital support staff		21 (1.7)
First responder		8 (0.6)
Community healthcare professional		32 (2.7)
Government / public service		124 (10.6)
Service industry (grocery, hardware, liquor)		110 (8.7)
Restaurant, bar, nightclub, entertainment industry		101 (8.6)
Education (primary/secondary/post-secondary		108 (9.2)
Other industries (energy/agriculture/natural resources/construction)		166 (14.2)
Other		458 (36.3)
**Chronic Health Conditions**	(n=1,940)	
Yes, current diagnosis		866 (44.6)

Additional File 2.

No current diagnosis	1074 (55.4%)

[1] Frequencies and percent are noted unless otherwise indicated. Prefer not to answer response options are excluded from data analyses and individual N reported.
[2] No respondents resided in Newfoundland and Labrador, Northwest Territories, Nunavut, or Yukon.
[3] Includes New Brunswick, Nova Scotia, and Prince Edward Island
[4] Percentage exceeds 100 as respondents were permitted to select up to 2 options of a list of 11 categories.
[5] Includes West Asian or Middle Eastern, African, Central/South American or Caribbean, Aboriginal/First Nations/Metis, and open-end Other
[6] Includes Hindu, Sikh, and open-end Other
[7] Includes response options "In a relationship, but not living together", "Living with a partner", and "Married"

Additional File 2.

SFigure 1. Pairwise Comparisons of Respondent Age, Gender, Income, and Education (n=1,996)

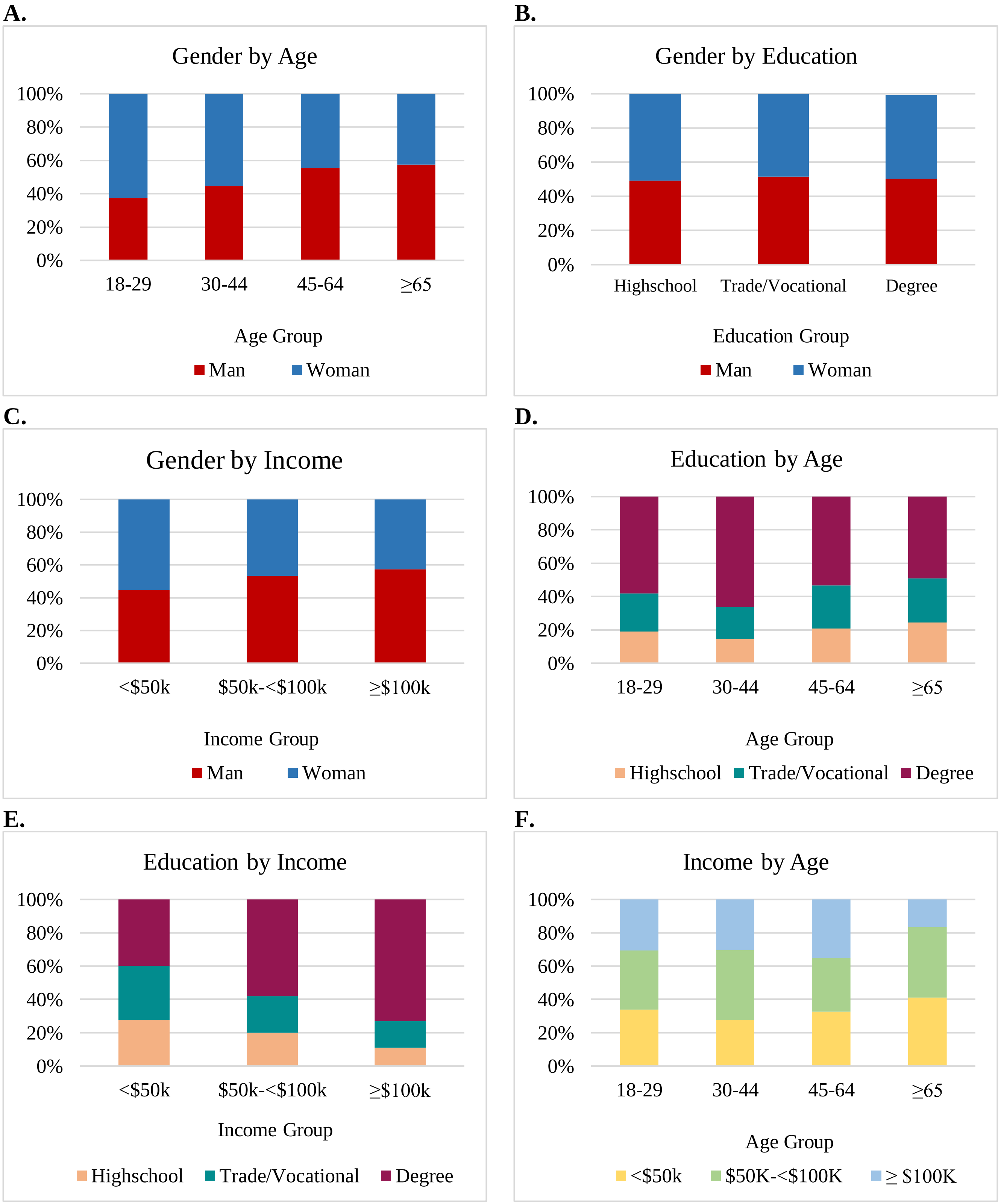

Abbreviations: Degree, Post-secondary undergraduate, graduate, or professional degree; High school, High school diploma, Collège d'enseignement général et professionnel, or less; Trade/Vocational, Trade, Vocational certification and/or some university or college
