# Supplementary material for: Public perceptions during the first wave of the COVID-19 pandemic in Canada: a demographic analysis of self-reported beliefs, behaviors, and information acquisition
Source: BMC Public Health. 2022 Apr 9;22:699. doi: 10.1186/s12889-022-13058-3 (PMC8994420; doi:10.1186/s12889-022-13058-3)
Supplement: Supplementary file 3 — Additional file 3: S1Figure. Perception of COVID-19 pandemic severity by age group. S2 Figure. Self-reported frequency of physical distancing by age group. S3 Figure. Self-reported duration that current levels of physical distancing could be maintained by age group. S4 Figure. Self-reported frequency of information seeking by age group. S5 Figure. Information sources selected as LEAST trustworthy by age group. S6 Figure. Information sources selected as MOST trustworthy by gender group. S7 Figure. Information sources selected as LEAST trustworthy by gender group. S8 Figure. Self-reported strategies used to verify information by education level. S9 Figure. Self-reported strategies used to verify information by age group. S10 Figure. COVID-19 topics searched for information about by age groug. [file 12889_2022_13058_MOESM3_ESM.pdf]

Additional File 3.

Supplemental data figures by age, gender, and education groups

SFigure 1 Perception of COVID-19 pandemic severity by age group (n=1992) 2

SFigure 2 Self-reported frequency of physical distancing by age group (n=1992) 3

SFigure 3 Self-reported duration that current levels of physical distancing could by maintained by age group (n=1984) 4

SFigure 4 Self-reported frequency of seeking information by age group (n=1982) 5

SFigure 5 Information sources selected as least trustworthy by age group (n=1897) 6

SFigure 6 Information sources selected as most trustworthy by gender group (n=1896) 7

SFigure 7. Information sources selected as least trustworthy by gender group (n=1882)........ 8

SFigure 8 Self-reported strategies used to verify information by education level (n=1974) 9

SFigure 9 Self-reported strategies used to verify information by age group (n=1996) 10

SFigure 10 COVID-19 topics searched for information by age group (n=1978)........ 11

Additional File 3.

SFigure 1. Perception of COVID-19 pandemic severity by age group (n=1992)

	Percent	OR (95% CIs)
**Very Serious**		
18–29	51.90%	1.00 (reference)
30–44	56.30%	1.20 (0.90–1.61)
45–64	60.90%	1.45 (1.10–1.92)
65+	75.10%	2.82 (2.09–3.81)
**Moderately Serious**		
18–29	32.60%	1.00 (reference)
30–44	25.00%	0.69 (0.50–0.94)
45–64	18.50%	0.47 (0.34–0.65)
65+	14.00%	0.34 (0.24–0.47)

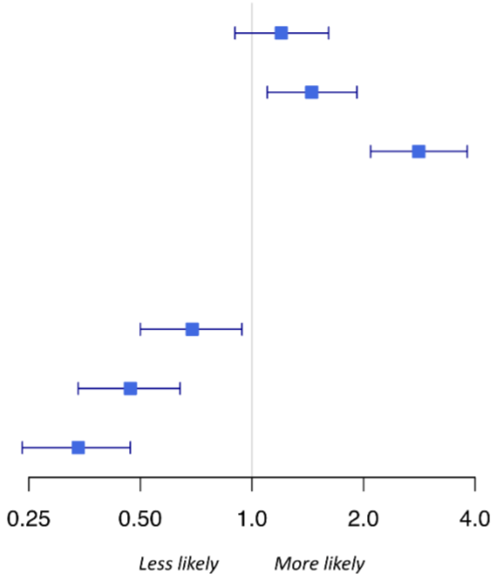

Abbreviations: CI, Confidence Intervals; OR, Odds Ratios
Legend: The x-axis utilizes a log scale. A 5-point question scale was used, and participants were given the options of Note Serious, Slightly serious, Somewhat serious, Moderately serious, and Very serious

Additional File 3.

SFigure 2. Self-reported frequency of physical distancing by age group (n=1992)

	Percent	OR (95% CIs)
**Always**		
18–29	51.30%	1.00 (reference)
30–44	59.00%	1.38 (0.92–2.08)
45–64	71.20%	2.37 (1.60–3.52)
65+	83.30%	4.82 (3.08–7.55)
**Often**		
18–29	40.00%	1.00 (reference)
30–44	35.00%	0.81 (0.53–1.23)
45–64	24.40%	0.48 (0.32–0.72)
65+	14.00%	0.24 (0.15–0.39)
**Sometimes**		
18–29	6.70%	1.00 (reference)
30–44	5.70%	0.83 (0.35–1.95)
45–64	3.00%	0.42 (0.17–1.02)
65+	1.70%	0.23 (0.08–0.70)

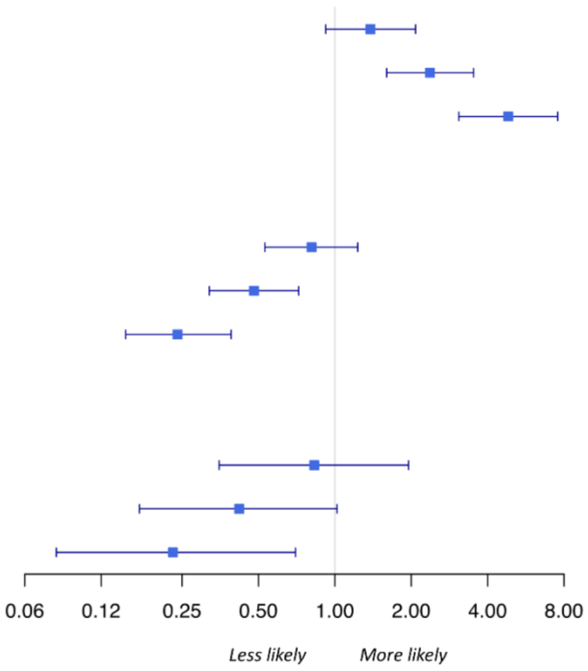

Abbreviations: CI, Confidence Intervals; OR, Odds Ratios
Legend: The x-axis utilizes a log scale.

Additional File 3.

SFigure 3. Self-reported duration that current levels of physical distancing could by maintained by age group (n=1984)

	Percent	OR (95% CIs)
**2 more weeks**		
18–29	7.9%	1.00 (reference)
30–44	5.9%	0.72 (0.41–1.26)
45–64	4.2%	0.51 (0.29–0.89)
65+	2.1%	0.23 (0.11–0.48)
**1 more month**		
18–29	19.4%	1.00 (reference)
30–44	14.8%	0.72 (0.50–1.05)
45–64	10.9%	0.50 (0.35–0.74)
65+	10.3%	0.48 (0.32–0.72)
**Longer than 6 months**		
18–29	33.0%	1.00 (reference)
30–44	32.4%	0.97 (0.72–1.32)
45–64	43.7%	1.58 (1.18–2.10)
65+	51.1%	2.14 (1.59–2.87)

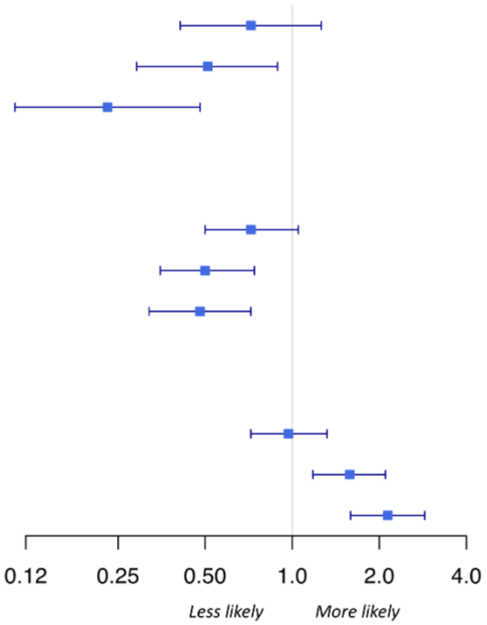

Abbreviations: CI, Confidence Intervals; OR, Odds Ratios
Legend: The x-axis utilizes a log scale.

Additional File 3.

SFigure 4. Self-reported frequency of seeking information by age group (n=1982)

	Percent	OR (95% CIs)
**Several times a day**		
18–29	17.10%	1.00 (reference)
30–44	25.60%	1.68 (1.17–2.42)
45–64	28.10%	1.91 (1.34–2.71)
65+	36.20%	2.77 (1.95–3.95)
**Once a day**		
18–29	34.00%	1.00 (reference)
30–44	37.20%	1.15 (0.85–1.55)
45–64	40.90%	1.34 (1.00–1.79)
65+	44.90%	1.58 (1.18–2.12)
**Every couple of days**		
18–29	29.20%	1.00 (reference)
30–44	19.80%	0.59 (0.42–0.82)
45–64	17.10%	0.49 (0.36–0.68)
65+	12.10%	0.33 (0.23–0.47)
**Once a week**		
18–29	16.20%	1.00 (reference)
30–44	12.90%	0.78 (0.52–1.16)
45–64	7.30%	0.42 (0.27–0.64)
65+	2.60%	0.14 (0.07–0.25)

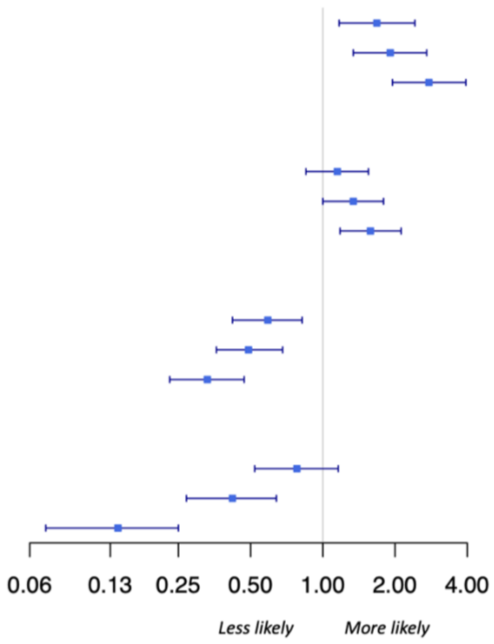

Abbreviations: CI, Confidence Intervals; OR, Odds Ratios
Legend: The x-axis utilizes a log scale.

Additional File 3.

SFigure 5. Information sources selected as least trustworthy by age group (n=1897)

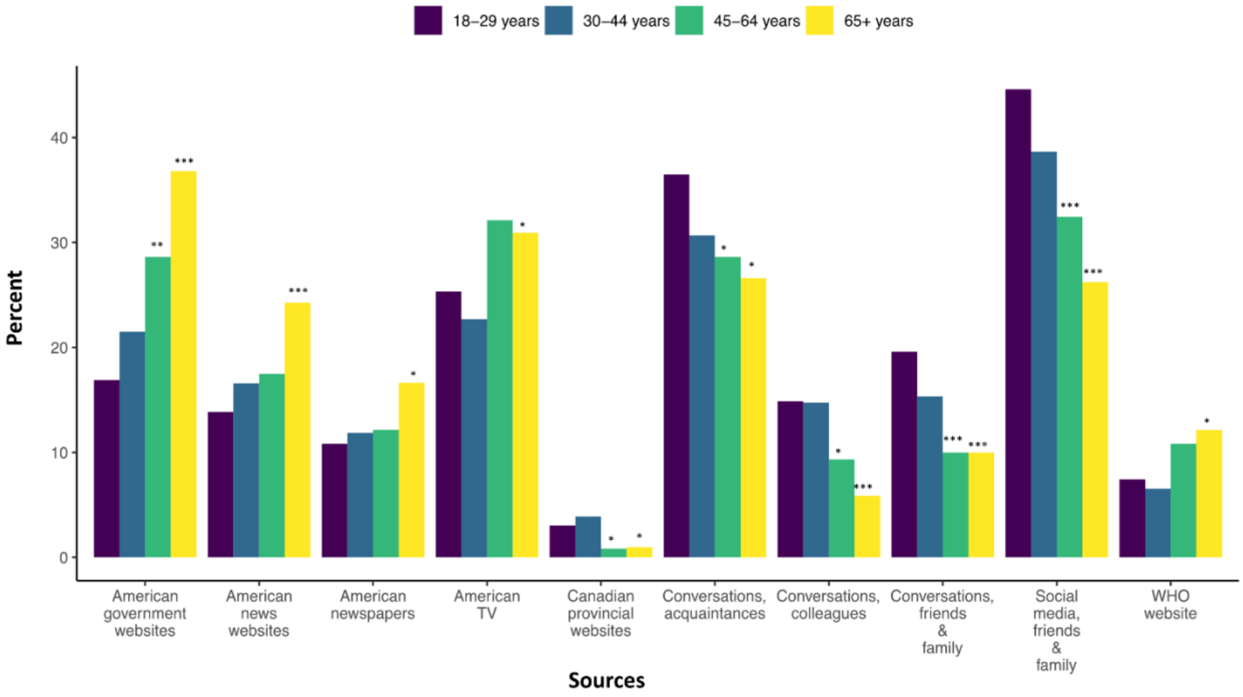

Abbreviations: TV, Television; WHO, The World Health Organization
Legend: Odds Ratios significance indicates category differs from the reference group (18–29-year-old) at *p<0.05, **p<0.01, and ***p<0.001.

Additional File 3.

SFigure 6. Information sources selected as most trustworthy by gender group (n=1896)

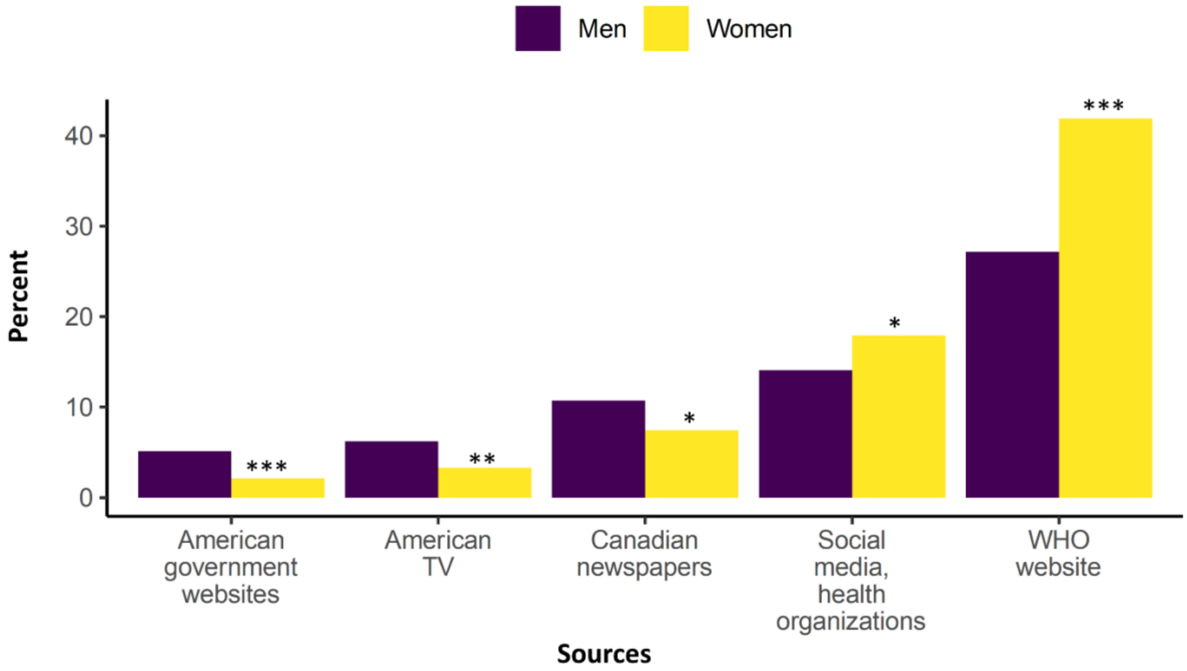

Abbreviations: TV, Television; WHO, The World Health Organization
Legend: Odds Ratios significance indicates category differs from the reference group (18–29-year-old) at *p<0.05, **p<0.01, and ***p<0.001.

Additional File 3.

SFigure 7. Information sources selected as least trustworthy by gender group (n=1882)

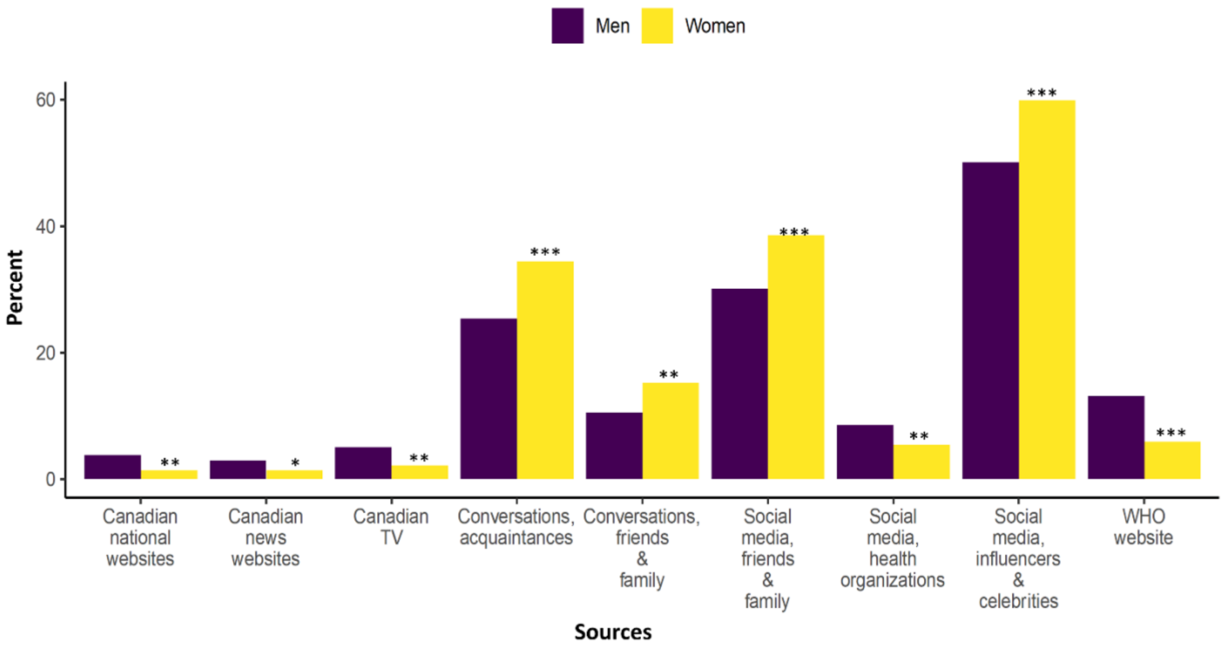

Abbreviations: TV, Television; WHO, The World Health Organization
Legend: Odds Ratios significance indicates category differs from the reference group (18–29-year-old) at *p<0.05, **p<0.01, and ***p<0.001.

Additional File 3.

SFigure 8. Self-reported strategies used to verify information by education level (n=1974)

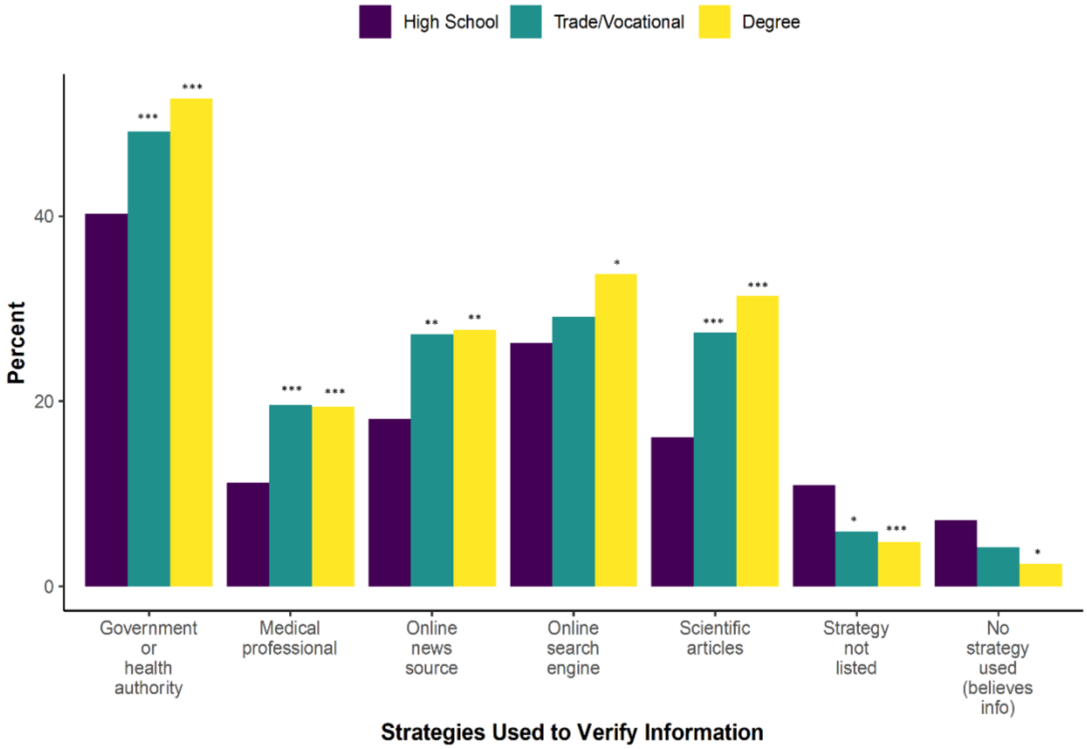

Abbreviations: Degree, Post-secondary undergraduate, graduate, or professional degree; High school, High school diploma. Collège d'enseignement general et professionnel, or less; Trade/Vocational, Trade, Vocational certification and/or some university or college

Legend: Odds Ratios significance indicates category differs from the reference group (18–29-year-old) at *p<0.05, **p<0.01, and ***p<0.001.

Additional File 3.

SFigure 9. Self-reported strategies used to verify information by age group (n=1996)

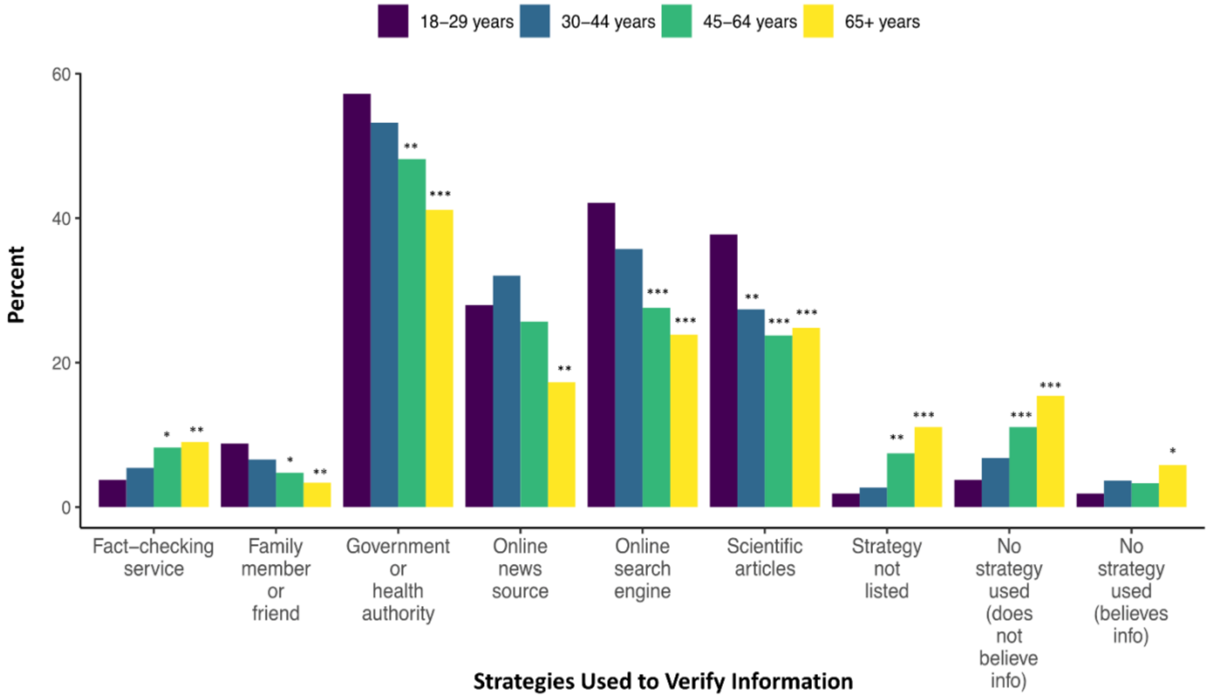

Legend: Odds Ratios significance indicates category differs from the reference group (18–29-year-old) at *p<0.05, **p<0.01, and ***p<0.001.

Additional File 3.

SFigure 10. COVID-19 topics searched for information by age group (n=1978)

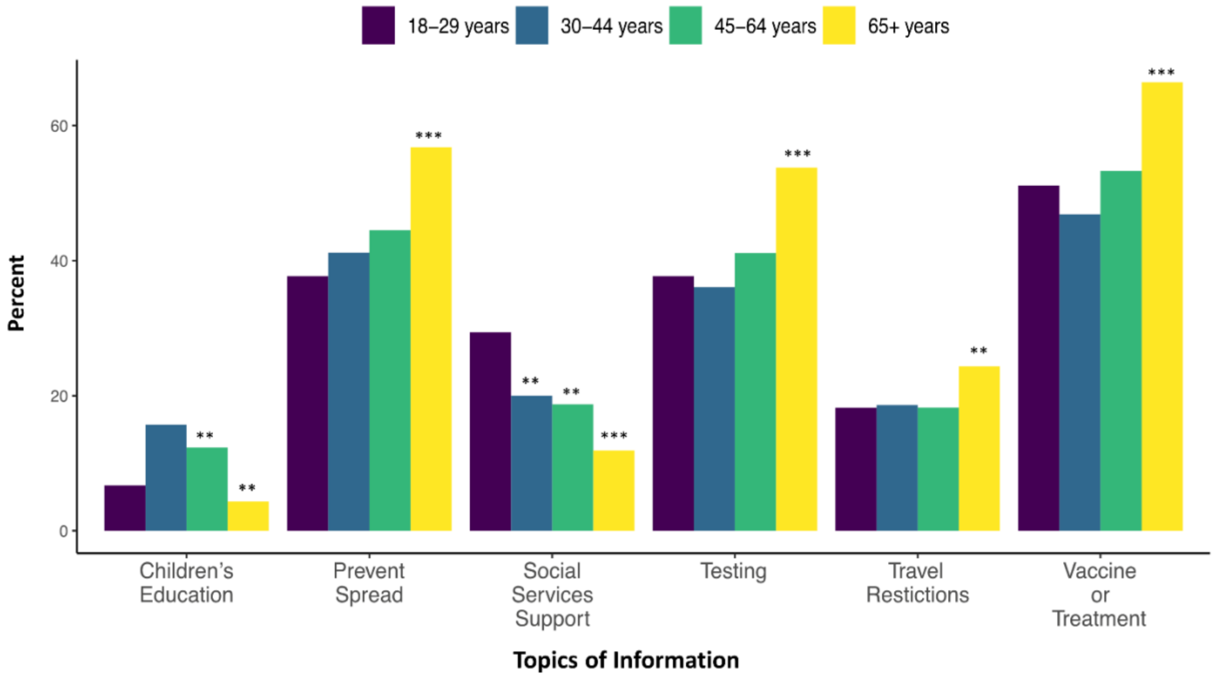

Legend: Odds Ratios significance indicates category differs from the reference group (18–29-year-old) at *p<0.05, **p<0.01, and ***p<0.001.
